# Insights into the deselenization of selenocysteine into alanine and serine† ‡
†Electronic supplementary information (ESI) available. See DOI: 10.1039/c5sc02528a
‡This paper is dedicated to Professor Donald Hilvert on the occasion of his 60th birthday.


**DOI:** 10.1039/c5sc02528a

**Published:** 2015-08-06

**Authors:** Shahar Dery, Post Sai Reddy, Linoy Dery, Reem Mousa, Rebecca Notis Dardashti, Norman Metanis

**Affiliations:** a Institute of Chemistry , The Hebrew University of Jerusalem , Jerusalem 91904 , Israel . Email: Metanis@mail.huji.ac.il

## Abstract

The development of native chemical ligation coupled with desulfurization has allowed ligation at several new ligation junctions. However, desulfurization also converts all cysteine residues in the protein sequence into alanine. Deselenization of selenocysteine, in contrast, selectively removes the selenol group to give alanine in the presence of unprotected cysteines. In this study we shed more light onto the deselenization mechanism of selenocysteine to alanine and provide optimized conditions for the reaction. The deselenization can be accomplished in one minute under anaerobic conditions to give alanine. Under aerobic conditions (oxygen saturation), selenocysteine is converted into serine.

## Abstract 2

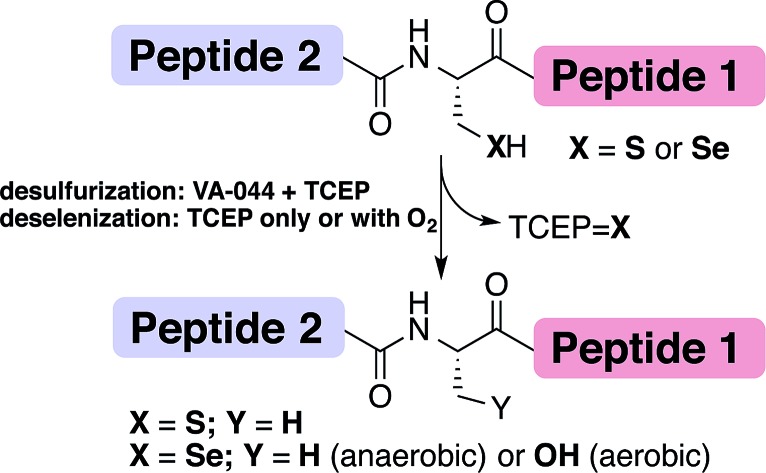
The deselenization of selenocysteine selectively removes the selenol group to give alanine under anaerobic conditions or serine under aerobic conditions (oxygen saturation).

## Introduction

**Fig. 1:**
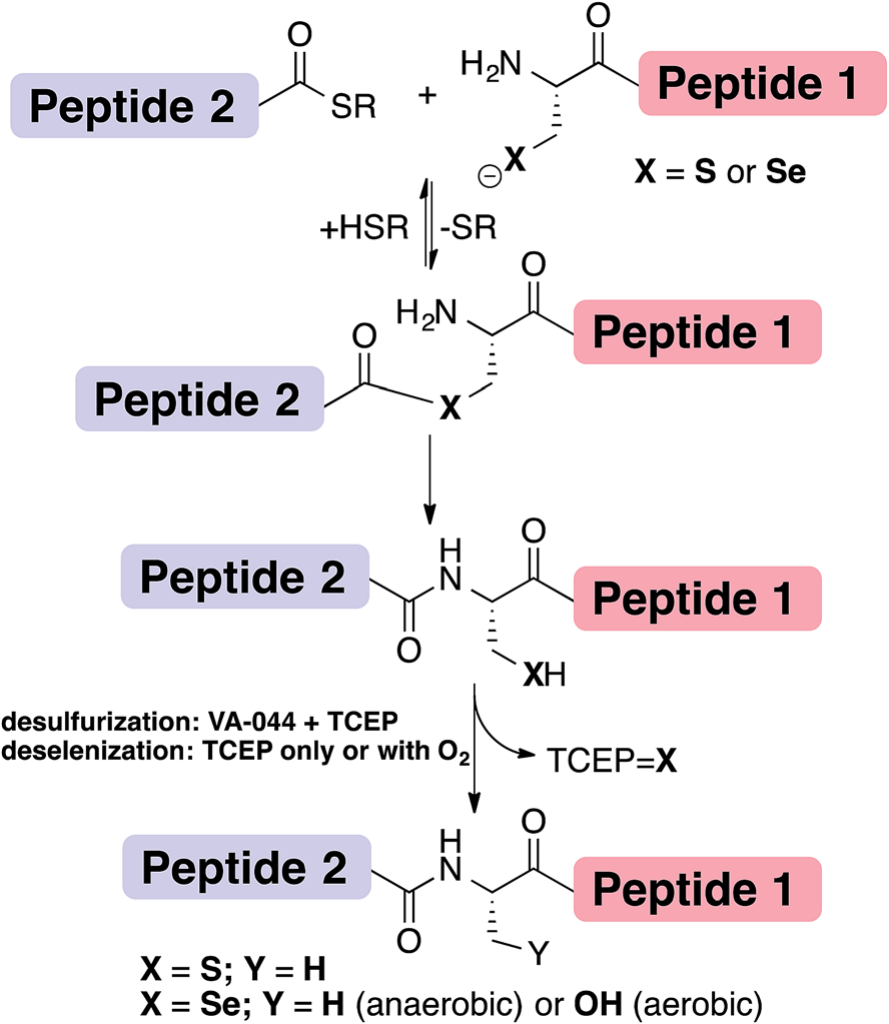
The NCL at Cys (X=S) or Sec (X=Se) and desulfurization/deselenization reactions. The desulfurization of Cys requires TCEP and the radical initiator VA-044 to give Ala, while deselenization of Sec requires only phosphine to give Ala (under anaerobic conditions) or Ser (under oxygen saturation conditions).

Chemical protein synthesis or semi-synthesis (CPS)^1^–^3^ allows for the preparation of proteins with exact control of their covalent structure. Hundreds of proteins up to ∼300 amino acids long^4^ have been prepared using a combination of solid-phase peptide synthesis (SPPS)^5^ and chemical ligation reactions, most notably through native chemical ligation (NCL) (Fig. 1).^1^,^6^ Since NCL requires a cysteine (Cys, C) residue at the ligation site, and because Cys is one of the least common amino acids in proteins, many research groups have developed methods that overcome this limitation by developing removable thiol auxiliaries,^7^–^12^ new ligation reactions,^13^–^16^ and NCL at residues with thiolated side-chains.^17^–^31^ One of the most robust strategies is the Yan and Dawson NCL/desulfurization approach, which extends the NCL to Xaa-Ala sites.^17^,^32^ Originally, Cys was desulfurized to Ala *via* hydrogenation over a metal catalyst (RANEY® Ni or H_2_/Pd/C).^17^ A less harsh, radical desulfurization was later developed by Wan and Danishefsky.^33^ In their method, based on the early work by Hoffmann and Walling,^34^,^35^ a radical initiator (VA-044) and phosphine (TCEP) performed global desulfurization under mild aqueous conditions.^33^ Despite the advantages of this approach, these methods not only desulfurize Cys at the ligation site, but also result in undesired desulfurization of native Cys residues anywhere in the protein. To overcome this limitation, an orthogonal protecting group on native Cys residues in the protein sequence can be used and later deprotected after the desulfurization step.^36^,^37^ However, the additional steps may lead to decreased yield of the overall reaction.^38^

An extension to NCL at selenocysteine was reported in 2001 by Raines, van der Donk and Hilvert (Fig. 1).^39^–^41^ Selenocysteine (Sec, U) is the 21^st^ encoded natural amino acid present in selenoproteins, including 25 known human selenoproteins.^42^–^44^ Lately, the presence of Sec in proteins has been harnessed to enhance folding^44^–^49^ and provide selective modification sites.^43^,^50^ We previously reported the traceless ligation of Cys peptides using selective deselenization of Sec to Ala using TCEP without an additional radical initiator.^51^ The deselenization of Sec was found to be chemoselective and enantioselective. As we previously suggested,^51^ an extension to the NCL/deselenization was reported for other residues with an additional selenol group on the side-chain,^32^,^52^–^54^ allowing for ligation at new residues while preserving native Cys elsewhere in the chain. A radical mechanism was proposed for the deselenization reaction,^51^ similar to the one proposed by Walling^34^ and Wan.^33^ However, this proposed mechanism has not been fully explored. Here, we report experimental evidence that supports the radical deselenization mechanism as well as optimized reaction conditions, in which Sec is deselenized to Ala within 1 minute. We also found that the formation of serine product (Sec to Ser conversion), which has previously been observed,^33^,^51^ is due to the presence of molecular oxygen. This reaction can be completely eliminated under anaerobic conditions or selectively and efficiently performed under saturated oxygen conditions. Notably, the latter observation may allow NCL at a Ser residue.

## Results and discussion

Our goal in this study was twofold: to gain insight into the mechanism of the deselenization reaction of Sec to Ala and to optimize it. We first tested the reaction of selenocystine with TCEP in D_2_O and followed the reaction progress using NMR. First, TCEP reduces selenocystine into selenocysteine, followed by deselenization (Fig. S1 and S2 in the ESI†). ^1^H-NMR confirmed the formation of mono-deuterated Ala (t_½_ = 30 min) with ∼90% conversion after 24 hours. The slow rate of the reaction can be attributed to the acidic conditions (pH∼1). The observed ^1^H-NMR shift confirms both the conversion of selenocysteine to mono-deuterated Ala (Fig. S3 and S4†), as well as conversion of TCEP to TCEP=Se and TCEP=O (Fig. S2–S7†). A reddish precipitate was formed within 2 weeks, indicating the formation of elemental selenium (Fig. S8†). ^31^P-NMR spectra revealed that only TCEP, TCEP=Se, and TCEP=O (Fig. S9–S12†) were present in solution, indicating that any intermediates formed during the deselenization reaction were extremely short-lived (such as **II** in Fig. 5).

**Fig. 2:**
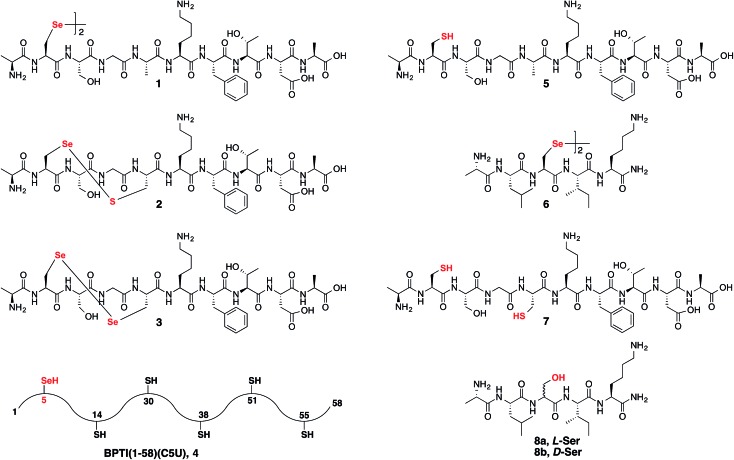
Synthetic peptides **1–8** used to study the deselenization reactions.

**Fig. 3:**
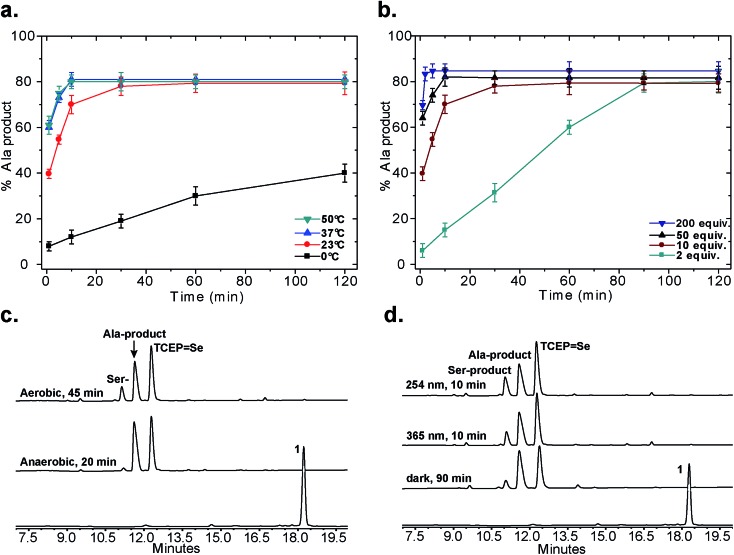
Control experiments for the deselenization of peptide **1**. (a) The effect of temperature; (b) the effect of TCEP concentration; (c) deselenization of peptide **1** under aerobic and anaerobic conditions (2 mM peptide **1**, 10 equiv. TCEP, 23 °C, pH 5, ambient light); (d) deselenization with irradiation (254 nm or 365 nm) or in the dark (2 mM peptide **1**, 10 equiv. TCEP, 23 °C, pH 5, aerobic). All peaks were characterized using LCMS, for further details on the conditions of each experiment see the ESI.† In (a) and (b) the s.d. values were calculated from experiments done in triplicate and the lines connecting the data points are shown only for clarity and do not represent a data fit.

Following our initial investigation, we synthesized a series of peptides (**1–8**, Fig. 2) and tested the reaction with TCEP under various conditions. Peptide **1** (AUSGAKFTDA), featuring a single Sec residue, was chosen as our control. Our starting conditions were phosphate buffer (100 mM PB, pH 5) at room temperature, as previously reported.^51^ We first investigated the effect of pH (pH 3, 5, and 7) and found pH 5 to be optimal.^51^ The reaction was also tested under different temperatures (0 °C, 23 °C, 37 °C and 50 °C). Both the yield and rate of deselenization increased with temperature from 0 °C to 37 °C. Further increase to 50 °C did not lead to a noticeable improvement (Fig. 3a).

We chose 23 °C to follow the changes in the deselenization reaction upon changing the parameters for subsequent control experiments. Increasing the concentration of TCEP (2, 10, 50 and 200 equiv.) over peptide **1** led to a faster reaction where with 200 equiv. TCEP we observed complete deselenization within 1 min (Fig. 3b and S13†). Interestingly, the optimized deselenization conditions, when applied to the Cys-containing analog peptide **5**, did not show any desulfurization, even after 24 h (Fig. S14†).

Under these ambient aerobic conditions, the Ser product was observed in significant amounts (20%) (Fig. 3c).^33^,^51^ In contrast, the proportion of the Ala product obtained was >95% when the reaction was carried out in degassed buffer in an anaerobic chamber (Fig. 3c, see also Fig. S13†). Previously we added dithiothreitol (DTT)^51^ to the reaction mixture. In the presence of DTT we observed a minor hydrolysis product that resulted from a N → Se acyl transfer shift as observed by other groups.^55^,^56^ This side-reaction was slightly inhibited by the addition of a bulky thiol (*t*BuSH) but was not completely eliminated. We therefore omitted any thiol additives from the following experiments.

Irradiation could in principle enhance the deselenization reaction by enhancing selenenyl radical formation, as suggested earlier for desulfurization reactions.^34^ Irradiation of the reaction vessel at 254 nm and 350 nm enhanced the reaction rate slightly (Fig. 3c *vs.* d). On the other hand, no significant change in the reaction rate was observed when the reaction was performed in the dark (Fig. 3d), indicating that other factors (*e.g.* temperature) play a more significant role in enhancing this reaction.

The rate of the deselenization reaction of peptide **1** increased significantly in the presence of the radical initiator VA-044, even when using a lower concentration of TCEP, and was completed within 1 min (10 equiv. VA-044 with 10 equiv. TCEP at 23 °C, Fig. S15†). In contrast, the desulfurization of the Cys-analog, peptide **5**, took 8 h to complete when VA-044 was present, even under more drastic conditions (10 equiv. VA-044, 200 equiv. TCEP at 37 °C, Fig. S16†). Interestingly, in the presence of 50 equiv. sodium ascorbate – a known radical quencher – the deselenization reaction of peptide **1** (with 10 equiv. TCEP) was almost completely halted even after 12 h (Fig. S17†), which further supports the proposed radical mechanism. Finally, Ollivier *et al.* recently suggested that TCEP=Se (a product of the deselenization reaction) completely inhibits the deselenization reaction.^55^ We found that even in the presence of 200 equiv. synthetic TCEP=Se the reaction proceeded smoothly, and the deselenized Ala product formed within 30 min (Fig. S18†). These results, together with the previous observations,^33^,^51^–^53^ support the proposed radical mechanism (Fig. 5).

Peptide **2**, with a Sec-X-X-Cys motif, was synthesized to test the selectivity of the deselenization reaction. As we preferred to omit any thiol additives to minimize hydrolysis side reactions, *vide supra*, we found that 2 equiv. TCEP is required for the selective deselenization of this peptide (Fig. S19†) as previously reported.^51^ The observed insignificant proportion of the desulfurization product is consistent with our previous results and may occur due to radical transfer from selenenyl to form a thiyl radical.^51^

The double deselenization^51^ of peptide **3** bearing a Sec-X-X-Sec motif (forming two Ala) was slowed due to the low redox potential of the diselenide bond in the UXXU motif.^59^ Therefore, in addition to 200 equiv. TCEP, we added 2 equiv. VA-044. The reaction proceeded smoothly to completion in 30 min (Fig. S20†). On the other hand, peptide **7**, the Cys analog of **3**, required 200 equiv. TCEP and 10 equiv. VA-044 at 37 °C to give the doubly desulfurized product after 8 h (Fig. S21†).

Finally, to verify the applicability of the selective deselenization reaction in a protein context, a seleno-analog of bovine pancreatic trypsin inhibitor (BPTI) in which Cys5 is substituted with Sec (BPTI(1–58)(C5U), peptide **4** in Fig. 2)^48^ was tested. This protein, containing 5 Cys residues and a single Sec, was selectively deselenized with 2 equiv. TCEP to give after ∼4 h the Ala-product; BPTI(1–58)(C5A) (Fig. S22†). The conditions used here required the addition of 4.2 equiv. DTT and 6 M guanidinium hydrochloride (GnHCl)^51^ to prevent protein folding and to ensure that Sec5 is completely solvent exposed.

**Fig. 4:**
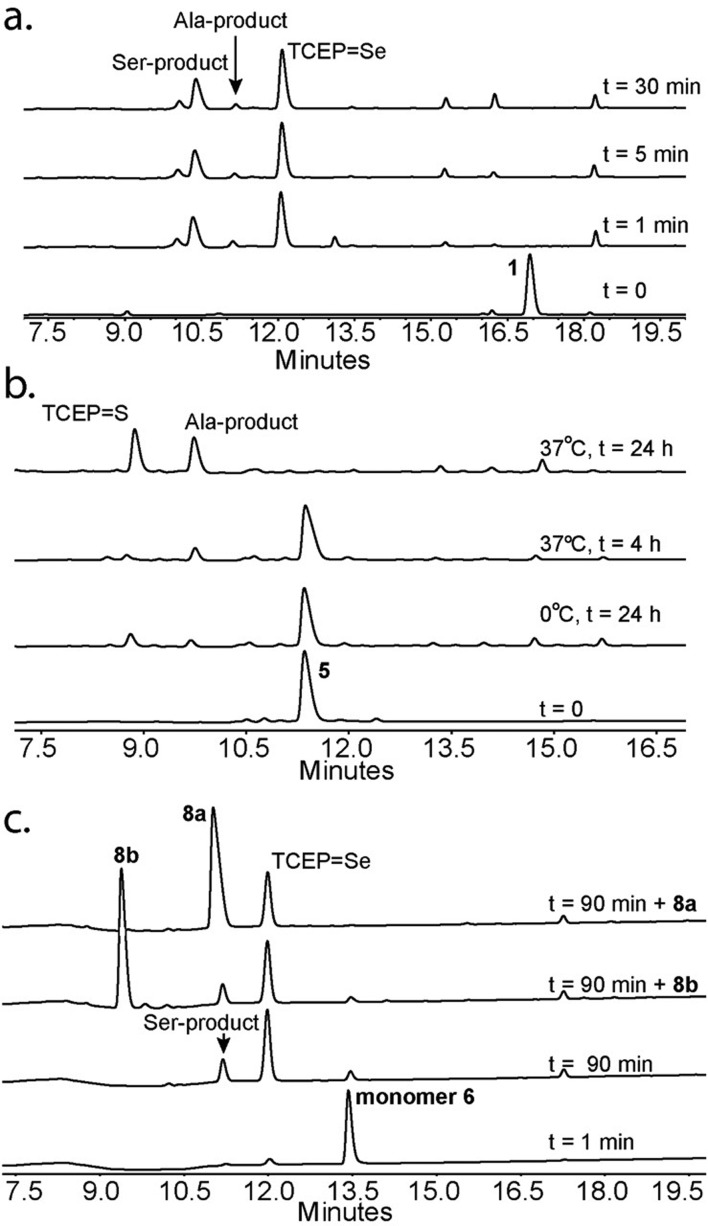
Conversion of Sec or Cys to Ser. (a) Deselenization of peptide **1** under oxygen-saturated conditions gives Ser as a major product. (b) Peptide **5** under varying conditions forms the Ala product. (c) Deselenization of peptide **6** under oxygen-saturated conditions forms the l-Ser product **8a**.

Next we focused on the production of the Ser product and wondered if it was possible to optimize conversion to the Ser product under aerobic conditions. We observed an increase in the formation of the Ser product when the reaction was exposed to a constant airflow (45% Ser in 10 min, Fig. S23†). Gratifyingly, when peptide **1** was treated with TCEP at 0 °C in oxygen-saturated buffer, the Ser product was observed as the major product within 5 min (Fig. 4a). This reaction is unique to Sec, as the reaction of Cys-containing peptide **5** under identical conditions showed no detectable Ser product and in the presence of VA-044 the Ala product was observed (Fig. 4b).

To confirm that the conversion of Sec to Ser is enantioselective, peptide **6** (ALUIK, isolated as a dimer) was prepared together with the two possible products containing l-Ser **8a** and d-Ser **8b** (Fig. 2). The conversion of Sec in peptide **6** to Ser was found to be enantioselective as judged by the retention time and co-injection of the reaction mixture with **8a** (Fig. 4c). This observation expands the NCL reaction to the Ser residue^16^,^57^,^58^ and could perhaps be equally expanded to other –OH containing amino acids.

**Fig. 5:**
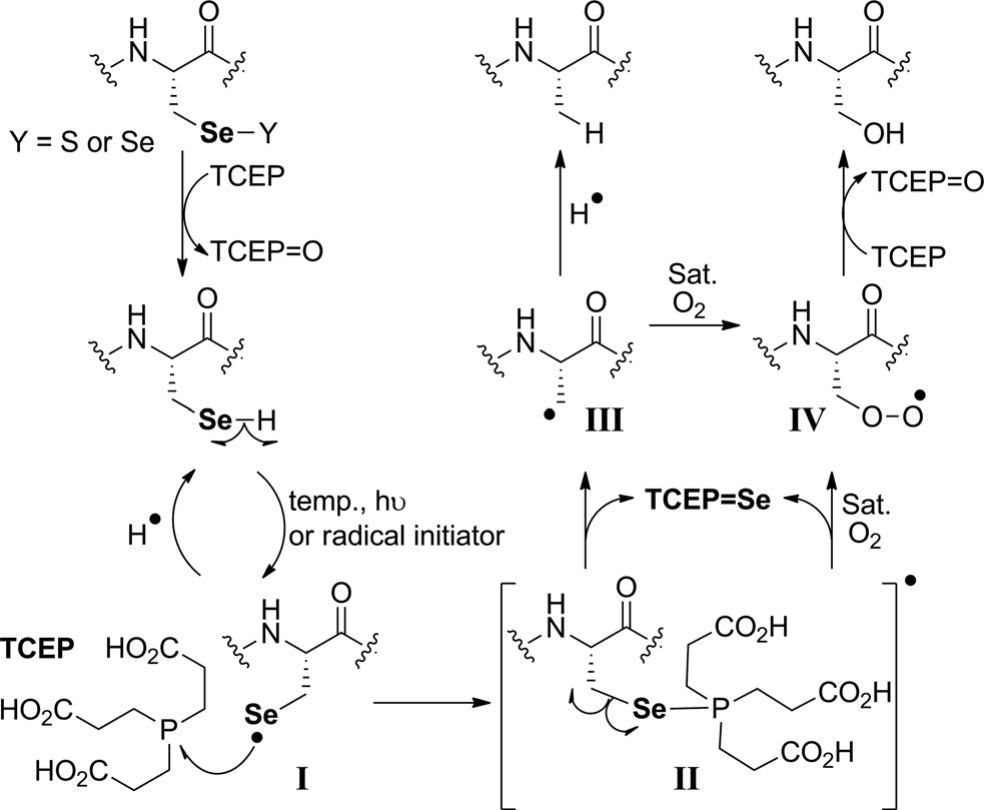
The proposed radical deselenization mechanism of Sec to Ala and Ser.

The proposed radical deselenization mechanism is shown in Fig. 5. First, a selenenyl radical is formed. The selenol (Se–H) bond is much weaker than thiol (S–H), with a bond dissociation energy (BDE) difference experimentally estimated to ∼13 kcal mol^–1^.^60^ As a result, the thiol group requires a radical initiator or elevated temperatures^61^,^62^ to form a thiyl radical (RS˙), whereas selenol readily forms the selenenyl radical (RSe˙, **I** in Fig. 5) at room temperature.^60^ Second, a direct attack of the selenenyl radical on the phosphorus atom forms the seleno-phosphoranyl radical **II** with expansion of the outer valence shell to accommodate nine electrons.^61^ As suggested earlier for the attack of a thiyl radical on phosphite (or phosphine),^61^ the selenenyl radical attack is extremely rapid and a low energy process. Subsequent homolytic C–Se bond cleavage (C–Se is ∼9.5 kcal mol^–1^ weaker than C–S) leads to the formation of an alkyl radical on the β-carbon **III**, which abstracts a hydrogen to form the Ala product.

The formation of the Ser product is interesting, as this product can be completely eliminated under anaerobic conditions or selectively formed under oxygen-saturated conditions. As we noted initially, the deselenization rate of Sec to Ala increases with increasing the temperature of the reaction, suggesting that the homolytic C–Se bond cleavage in the seleno-phosphoranyl radical (**II**) becomes more favored. On the other hand, decreasing the temperature could slow the C–Se bond cleavage and allow other reactions, such as the attack of the diradical oxygen molecule (if present), to take precedence. The inability of peptide **5** to form the Ser product, even under oxygen-saturated conditions, is consistent with our proposed mechanism, in which molecular oxygen is converted to a radical species *via* direct contact with the seleno-phoshoranyl radical **II**. This radical intermediate gives the peroxy-radical **IV**, which is reduced by TCEP to give Ser. In contrast, the alkyl radical on the β-carbon **III** (a common species in desulfurization and deselenization reactions) has little or no effect on molecular oxygen, thus no Cys to Ser conversion is observed with peptide **5**. The driving force of the deselenization reactions is the formation of a strong P=Se bond in TCEP=Se.

## Conclusions

In this work, we have optimized both the selective deselenization reaction and provided considerable experimental evidence to support the previously suggested radical mechanism.^51^ Under the conditions examined, the deselenization reaction can be completed in 1 min, the fastest deselenization ever reported, which may also inhibit side reactions. In addition to these observations, we showed that selenocysteine can be converted selectively to either alanine or serine simply by varying the oxygen content of the reaction.

We envision that selenocysteine modification reactions such as the deselenization reactions presented in this study will find future utility in chemical protein synthesis. This will enable the use of native cysteines for some ligation sites and selenocysteines at sites in which a non-chalcogen containing amino acid is desired.

## Supplementary Material

Supplementary information
