# Supplementary material for: Insights into the deselenization of selenocysteine into alanine and serine
Source: Chem Sci. 2015 Aug 6;6(11):6207–12. doi: 10.1039/c5sc02528a (PMC6054048; doi:10.1039/c5sc02528a)
Supplement: Supplementary file 1 [file SC-006-C5SC02528A-s001.pdf]

Electronic Supplementary Material (ESI) for Chemical Science.
This journal is © The Royal Society of Chemistry 2015

Supporting Information

Insights into the Deselenization of Selenocysteine into Alanine and Serine

Shahar Dery, Post Sai Reddy, Linoy Dery, Reem Mousa, Rebecca Notis Dardashti and Norman Metanis*

Experimental Section:

Supporting Materials and Methods. Buffers were prepared using MilliQ water. NaH_2PO_4 and Na_2HPO_4 were purchased from Sigma-Aldrich. Deuterated oxide (D_2O, $CDCl_3$), 2,2'-dithiobis(5-nitropyridine) (DTNP) and tris(2-carboxyethyl)phosphine (TCEP), Trifluoromethanesulfonic acid (TfOH), Methyl sulfide (DMS), ethanedithiol (EDT), triisopropylsilane (TIS) and thioanisole were purchased from Sigma-Aldrich (Rehovot, Israel). ^{1}H- and ^{31}P-NMR spectra were recorded on a Bruker 400 or 500 MHz instruments with chemical shifts reported in ppm relative to the residual deuterated solvent. All Boc-amino acids were obtained from CS Bio Co. (Menlo Park, CA), with the following side chain protecting groups: Asp(OBzl), Glu(OBzl), Ser(OBzl), Thr(OBzl), Cys(4-Me-Bzl), Lys(2Cl-Z), Tyr(2Br-Z), (Bzl = benzyl; 4-Me-Bzl = 4-methylbenzyl; 2Cl-Z = 2-chlorobenzyloxycarbonyl; 2Br-Z = 2-bromobenzyloxycarbonyl). Boc-L-Ala-Pam resin was obtained from Iris Biotech GmbH (Germany). All Fmoc-amino acids were obtained from CS Bio Co. (Menlo Park, CA) or Matrix Innovation (Quebec City, Canada), with the following side chain protecting groups: Arg(Pbf), Asp(OtBu), Glu(OtBu), Ser(tBu), Thr(tBu), Cys(Trt), Lys(Boc), Tyr(tBu), Asn(Trt). (Pbf = 2,2,4,6,7-pentamethyl-2,3-dihydrobenzofuran-5-sulfonyl). Fmoc-*L*-Ala-WANG resin was obtained from Iris Biotech GmbHand and Fmoc-Rink amide resin from Matrix Innovation. 1-[Bis(dimethylamino)methylene]-1H-1,2,3-triazolo[4,5-b]pyridinium 3-oxide hexafluorophosphate (HATU) and Ethyl cyano(hydroxyimino)acetate (OxymaPure) were generous gift from Luxembourg Biotechnologies Ltd. (Rehovot, Israel). All solvents: *N,N*-dimethylformamide (DMF), dichloromethane, and acetonitrile (ACN), *N,N*-diisopropylethyl amine (DIEA), piperidine (Pip) purchased from Bio-Lab (Jerusalem, Israel) and were peptide synthesis, HPLC or ULC-grade . Trifluoroacetic acid (TFA) was a generous gift from Halocarbon Products (River Edge, NJ).

High Performance Liquid Chromatography (HPLC). Analytical reversed-phase HPLC (RP-HPLC) was performed on a Waters UPLC H-Class with 220 nm UV detection using a XSelect C18 column (3.5 µm, 130 Å, 4.6 × 150 mm). Preparative and Semi-preparative RP-HPLC was performed on a Waters LCQ150 system using a XBridge C8 column (5 µm, 10 × 150 mm) or XSelect C18 column (5 µm, 130 Å, 30 × 250 mm). Linear gradients of acetonitrile in water with 0.1% TFA were used for all systems to elute bound peptides. The flow rates were 1 mL/min (analytical), 3.35 mL/min (Semi-preparative), and 20 mL/min (preparative).

UV Irradiation. Reactions under UV irradiations were performed in a CN-6 Darkroom with a 230 V ~50/60 Hz UV lamp containing a 2x6W 254 nm tube and 2x6W 365 nm tube (Viber Lourmant, France).

Mass Spectrometry. Electrospray ionization MS was performed on LCQ Fleet Ion Trap mass spectrometer instrument (Thermo Scientific). Peptide masses were calculated from the experimental mass to charge (*m/z*) ratios from all of the observed multiply charged species of a peptide.

Synthesis of protected Boc-Sec(Mob)-OH and Fmoc-Sec(Mob)-OH. The synthesis was performed as described elsewhere.[1, 2]

Peptide synthesis.

Boc-SPPS. Peptides (**1-3**) were prepared by manual solid-phase peptide synthesis (SPPS) on a Boc-*L*-Ala-PAM resin, typically on a 0.3 mmol scale using the *in situ* neutralization/HCTU activation procedure for Boc-SPPS.[3] The peptide coupling was carried out with 5-fold excess of activated amino acid for 20 min. Boc-Sec(Mob)-OH coupling was carried out manually using a DIC/Oxyma activation method,[1] using Boc-Sec(Mob)-OH (0.5 mmol in 2 mL of 50% DCM/DMF, 1.67 eq.) and activated with DIC (0.5 mmol, 1.67 eq.) in the presence of Oxyma (0.52 mmol, 1.73 eq.) at 0 °C for 5 min. The Boc group on resin-bound peptide was deprotected, neutralized with DIEA (2 × 1 min), and washed with DMF. The activated Sec was then added to the resin, and the mixture was shaken at room temperature for at least 1 h. After chain assembly, peptide side-chain deprotection and cleavage from the resin (200 mg) was carried out by standard TfOH protocol[4] with an additional 50 mg 2,2'-dithiobis(5-nitropyridine) (DTNP)[5]. The crude peptide products were precipitated and washed with cold anhydrous ether, dissolved in aqueous acetonitrile lyophilized and later purified by RP-HPLC.

Fmoc-SPPS. Peptides (**5-8**) were prepared manually or by automatic peptide synthesizer (CS136XT, CS Bio Inc. CA) on Fmoc-*L*-Ala-WANG resin, typically on a 0.25 mmol scale. Fmoc deprotection was carried out with 20% piperidine in DMF (5 min × 2). Fmoc-amino acids (2 mmol in 5 mL DMF) activated with HATU (2 mmol in 5 mL DMF) and DIEA (4 mmol in 5 mL DMF) for 5 min and allowed to couple for 30 min, with constant shaking. The resulting resins were washed with DMF (x3) and DCM (x3) and dried.

For cleavage of 200 mg resin, 13 mL cleavage cocktail was prepared (94% TFA:1.5% H_2O:1.5% EDT/:1.5% TIS:1.5% thioanisole). If Sec was present 50 mg of DTNP was included. The mixture was added to the peptide-resin and shaken for 3 h. The resin was removed by filtration, and washed twice with neat TFA. TFA was removed by N_2 bubbling, followed by addition of cold ether to precipitate the peptide. After centrifugation (5000 rpm, 5 min) and decanting ether, peptides were dissolved in 0.1% TFA in H_2O and lyophilized. The resulting crude peptide was dissolved in aqueous acetonitrile or phosphate buffer pH 7 and treated with DTT, and purified by preparative RP-HPLC.

Synthesis of TCEP=Se: TCEP=Se is a product of the deselenization reaction, and was prepared for control experiments from Se powder (0.1 g, 1.27 mmol) and TCEP (50 mg, 0.17 mmol) in D_2O. The ^{1}H- and ^{31}P-NMR are shown in Figures S7 & S10.

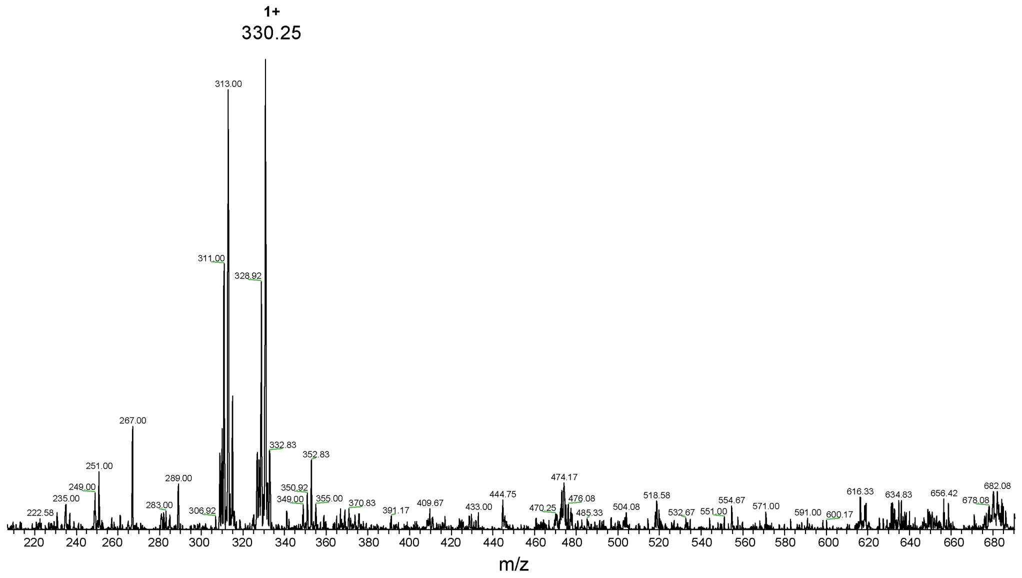

ESI-MS of synthetic TCEP=Se. Mass calc. 329.98, found 329.25.

Deselenization of selenocysteine by TCEP followed by NMR

Selenocystine (4 mg, 12 μmol) and TCEP (13.5 mg, 47μmol) were dissolved in 200 μL of D_2O. The progress of the reaction was followed as a function of time by ^{1}H- and ^{31}P-NMR (5, 20, 30 min, 1 d and 4 d and after 2 weeks) (See Figures S1-S7 and S9-S12).

Optimized procedure for deselenization

In an anaerobic chamber (Coy Laboratory Products, MI), a solution of 400 mM TCEP in degassed 100 mM phosphate buffer, pH 5 was prepared. Peptide **1** was added to give 2 mM solution. Reaction was left at room temperature without shaking and was complete in 1-2 min.

Characterization of peptide 1-8

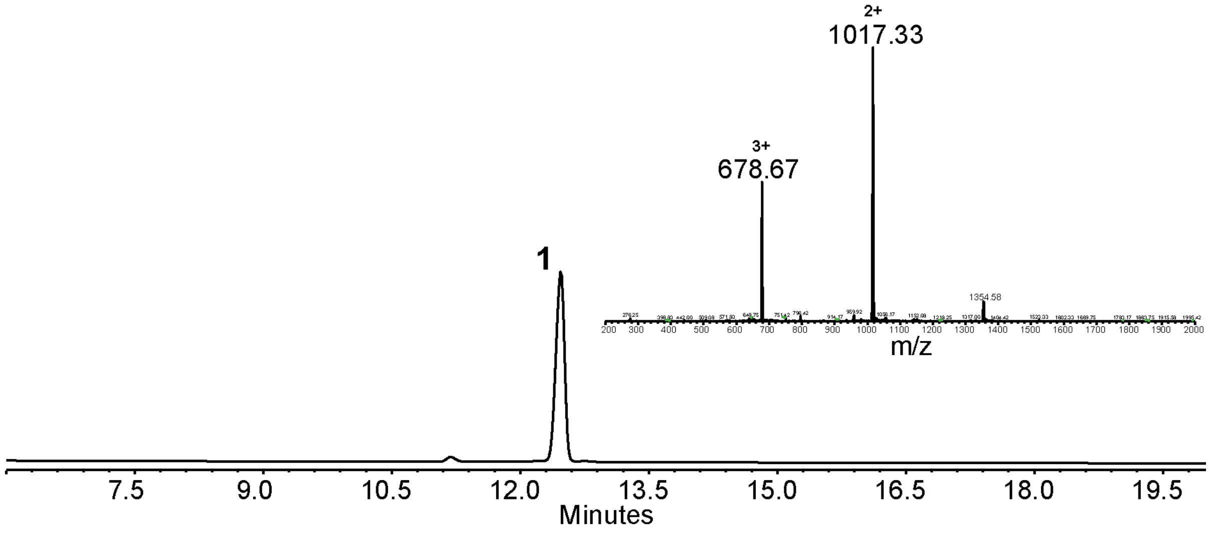

Purified peptide **1** with ESI-MS. Mass calc. 2032.72, found 2032.84.

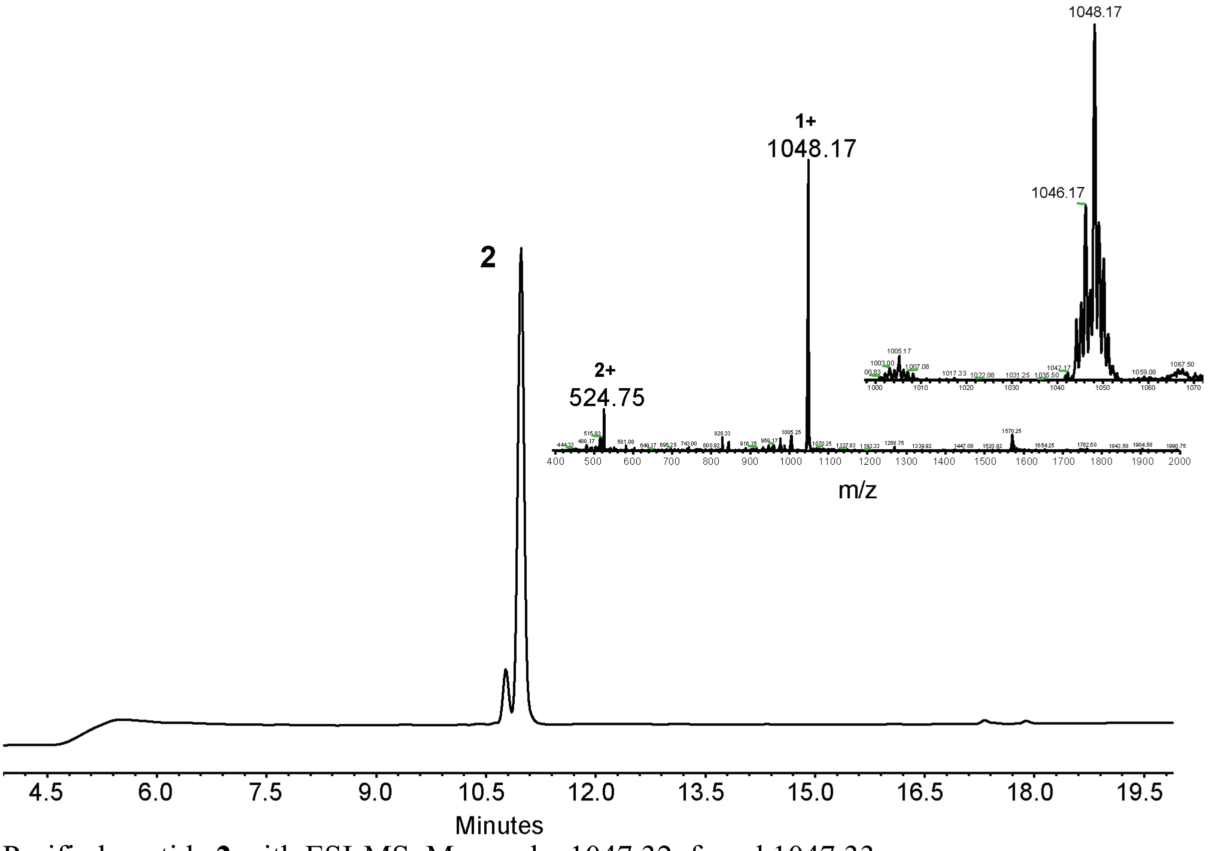

Purified peptide **2** with ESI-MS. Mass calc. 1047.32, found 1047.33.

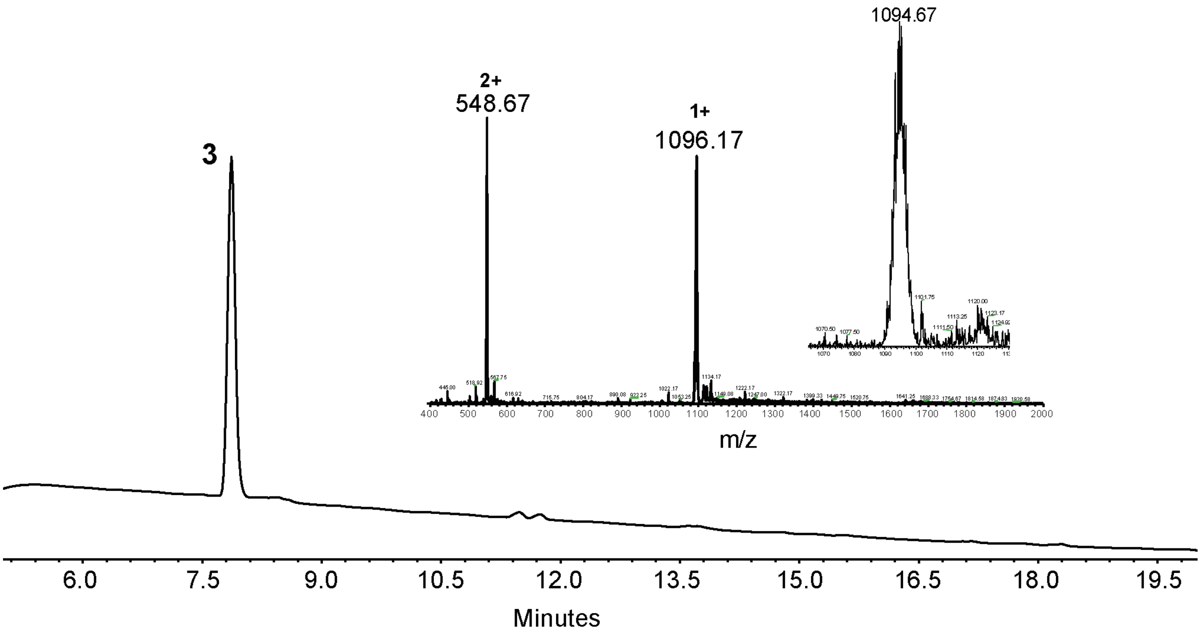

Purified peptide **3** with ESI-MS. Mass calc. 1095.27, found 1095.25.

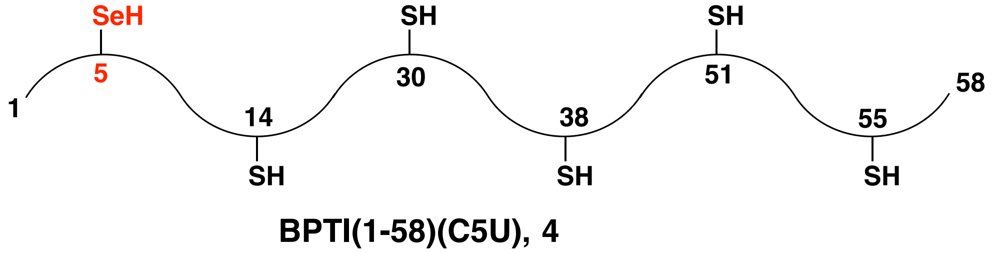

BPTI(1-58)(C5U) (peptide **4**) was prepared through NCL as previously reported,[6] and was used without further purification for our deselenization reactions. Mass for fully reduced calc. 6546.4, found 6546.1 Da (See Figure S23).

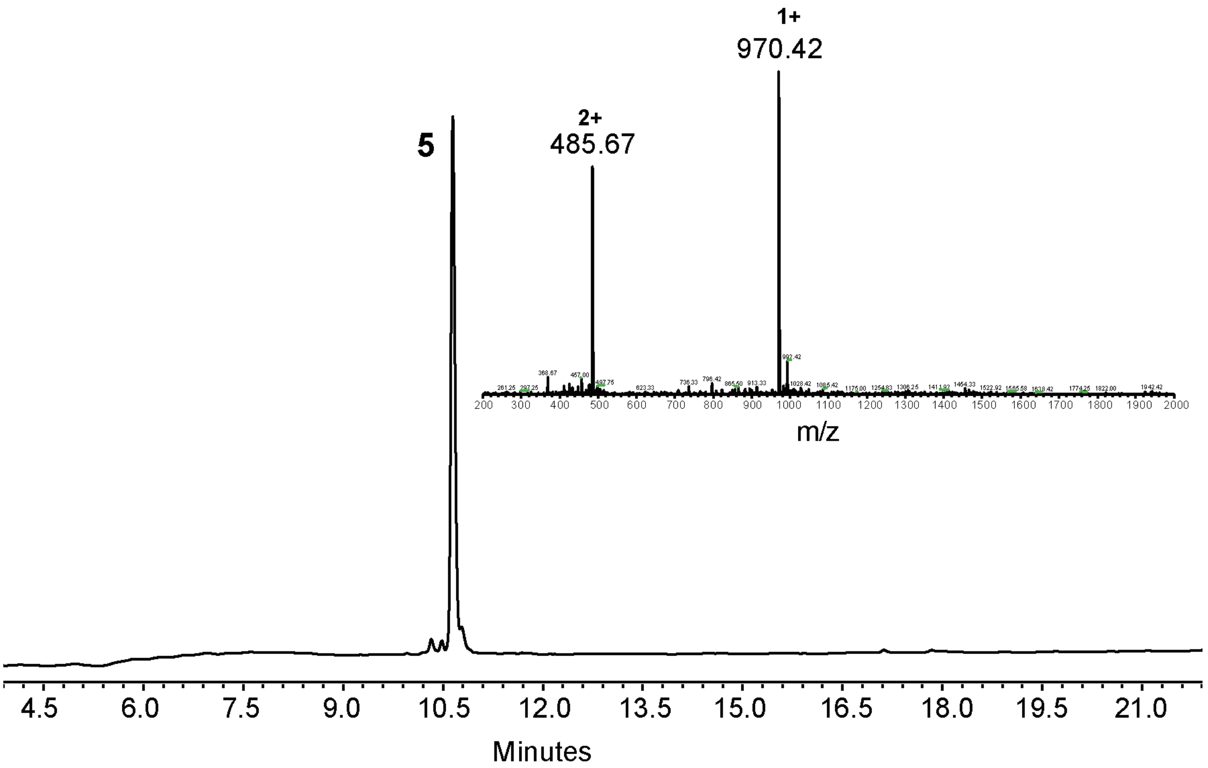

Purified peptide **5** with ESI-MS. Mass calc. 969.42, found 969.38.

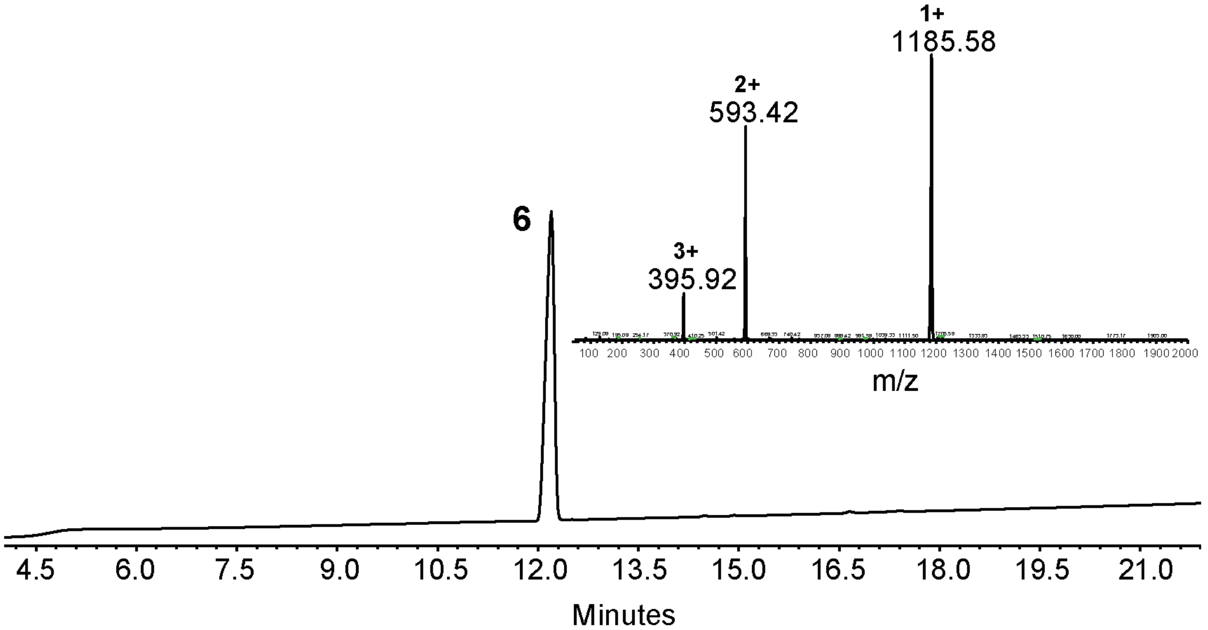

Purified peptide **6** with ESI-MS. Mass calc. 1184.55, found 1184.73.

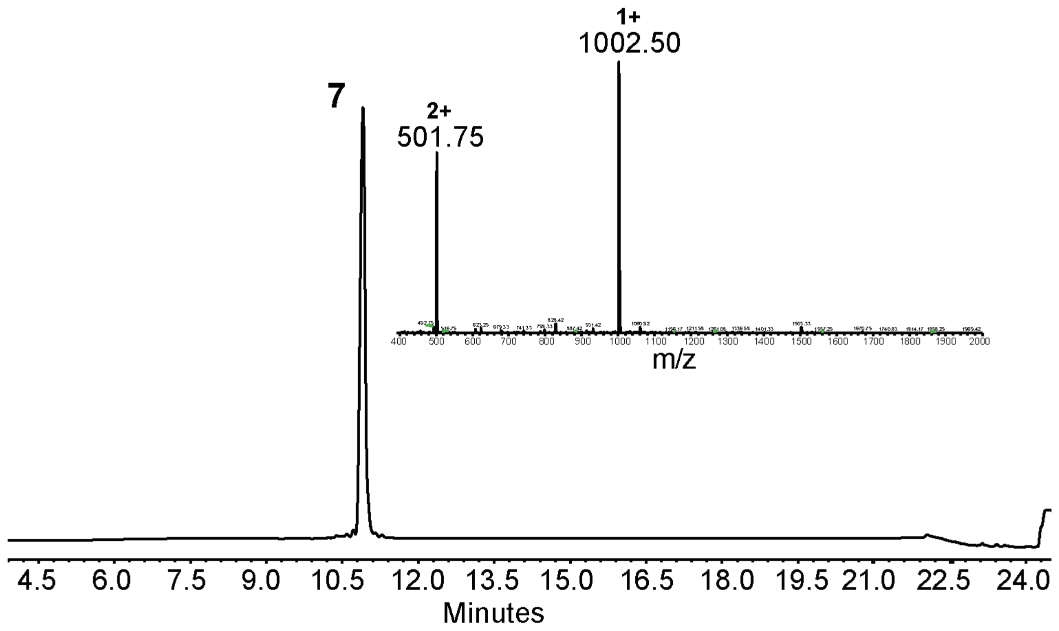

Purified peptide **7** with ESI-MS. Mass calc. 1001.39, found 1001.50.

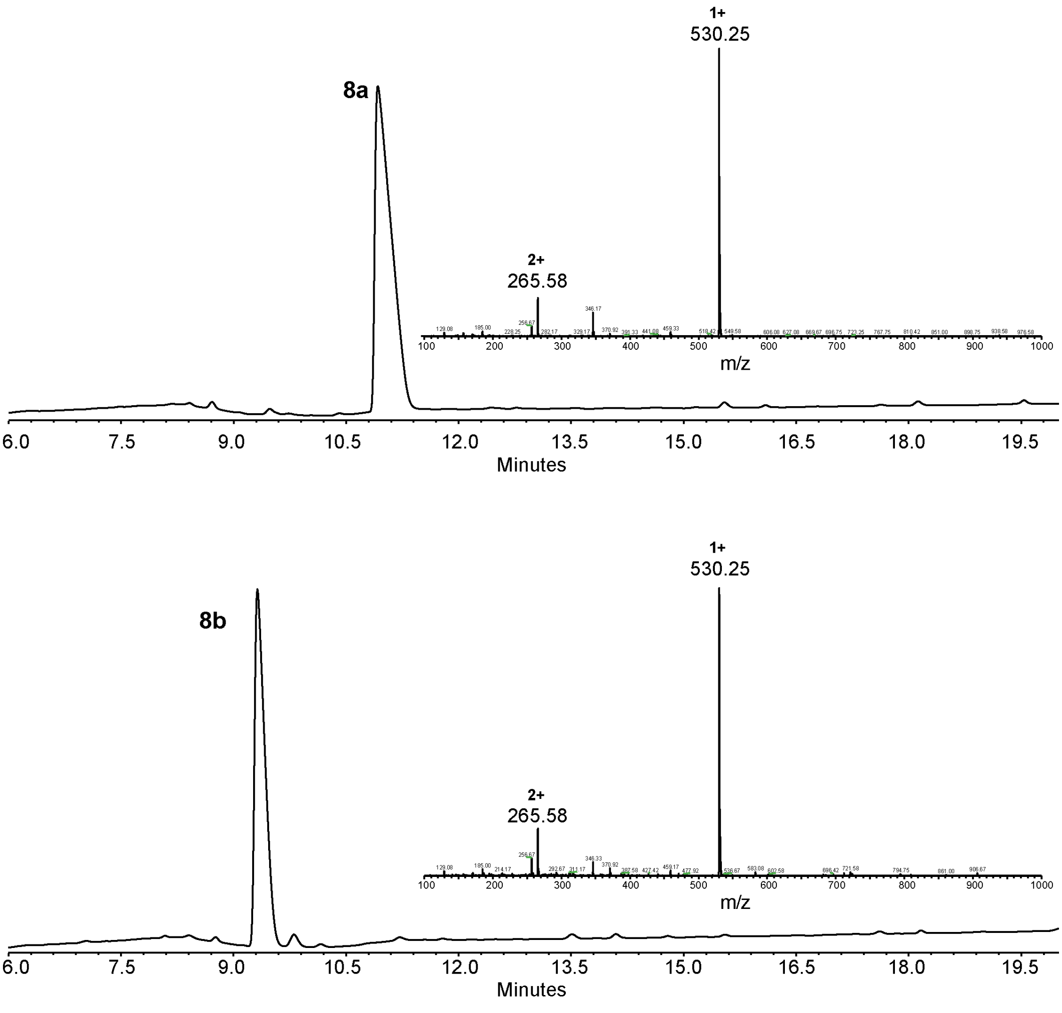

Purified peptide *L*-Ser-peptide **8a** and *D*-Ser-peptide **8b** (retention time difference >1 min) with ESI-MS. Mass calc. 529.36, found 529.21.

Summary of peptide 1 (AUSGAKFTDA) **deselenization experiments**

All experiment contain: 2 mM peptide **1** in 100 mM phosphate buffer, pH 5, under aerobic, ambient light and 23ºC unless otherwise specified.

pH and thiol additive control experiments:

1) 2 equiv. TCEP; **2**) 10 equiv. TCEP; **3**) 50 equiv. TCEP; **4**) 200 equiv. TCEP; **5**) 10 equiv. TCEP, 10 equiv. DTT, pH 7; **6**) 10 equiv. TCEP, 10 equiv. *t*-BuSH, pH 7; **7**) 10 equiv. TCEP, 10 equiv. *t*-BuSH, pH 3; **8**) 200 equiv. TCEP, anaerobic.

Temperatures control experiments:

1) 10 equiv. TCEP, 23ºC; **2**) 10 equiv. TCEP, 37ºC; **3**) 10 equiv. TCEP, 0ºC; **4**) 10 equiv. TCEP, 50ºC.

Radical inhibiting conditions:

1) 10 equiv. TCEP, 50 equiv. sodium ascorbate; **2**) 50 equiv. TCEP, 200 equiv. TCEP=Se; **3**) 10 equiv. TCEP, dark.

Radical promoting conditions:

1) 10 equiv. TCEP, 254 nm; **2**) 10 equiv. TCEP, 365 nm; **3**) 50 equiv. TCEP, aerobic (air bubbling); **4**) 10 equiv. TCEP, 10 equiv. VA-044, anaerobic; **5**) 50 equiv. TCEP, oxygen bubbling, 0ºC.

Peptide 2 (AUSGCKFTDA) **deselenization experiment**

All experiment contain: 2 mM peptide **2** in 100 mM phosphate buffer, 2 equiv. TCEP, pH 5, anaerobic chamber, ambient light and 23°C.

Summary of peptide 3 (AUSGUKFTDA) **deselenization experiments**

All experiment contain: 2 mM peptide **3** in 100 mM phosphate buffer, pH 5, under aerobic, ambient light and 23°C unless otherwise specified.

1) 200 equiv. TCEP; **2**) 200 equiv. TCEP, 2 equiv. VA-044, anaerobic chamber.

Deselenization experiment of BPTI(1-58)(C5U), peptide 4

~0.5 mM BPTI(1-58)(C5U) with 4.2 equiv DTT, 2 equiv. TCEP, in 100 mM phosphate buffer containing 6 M GnHCl, pH 5, in anaerobic chamber, ambient light and 23°C

Summary of peptide 5 (ACSGAKFTDA) **desulfurization experiments**

All experiment contain: 2 mM peptide **5** in 100 mM phosphate buffer, pH 5, under aerobic, ambient light and 23°C unless otherwise specified.

1) 200 equiv. TCEP; **2**) 200 equiv. TCEP, 10 equiv. VA-044, anaerobic chamber, 37°C; **3**) 200 equiv. TCEP, 10 equiv. VA-044, O_2 bubbling, 37°C; **4**) 200 equiv. TCEP, 10 equiv. VA-044, O_2 bubbling, 0°C.

Summary of peptide 6 (ALUIK) **deselenization experiments**

All experiment contain: 2 mM peptide **6** in 100 mM phosphate buffer, pH 5, under aerobic, ambient light and 23°C unless otherwise specified.

1) 10 equiv. TCEP, O_2 bubbling, 0°C.

Summary of peptide 7 (ACSGCKFTDA) **desulfurization experiments**

All experiment contain: 2 mM peptide **7** in 100 mM phosphate buffer, pH 5, under aerobic, ambient light and 23°C unless otherwise specified.

1) 200 equiv. TCEP; **2**) 200 equiv. TCEP, 10 equiv. VA-044, anaerobic chamber, 37°C.

Peptide 1 deselenization products

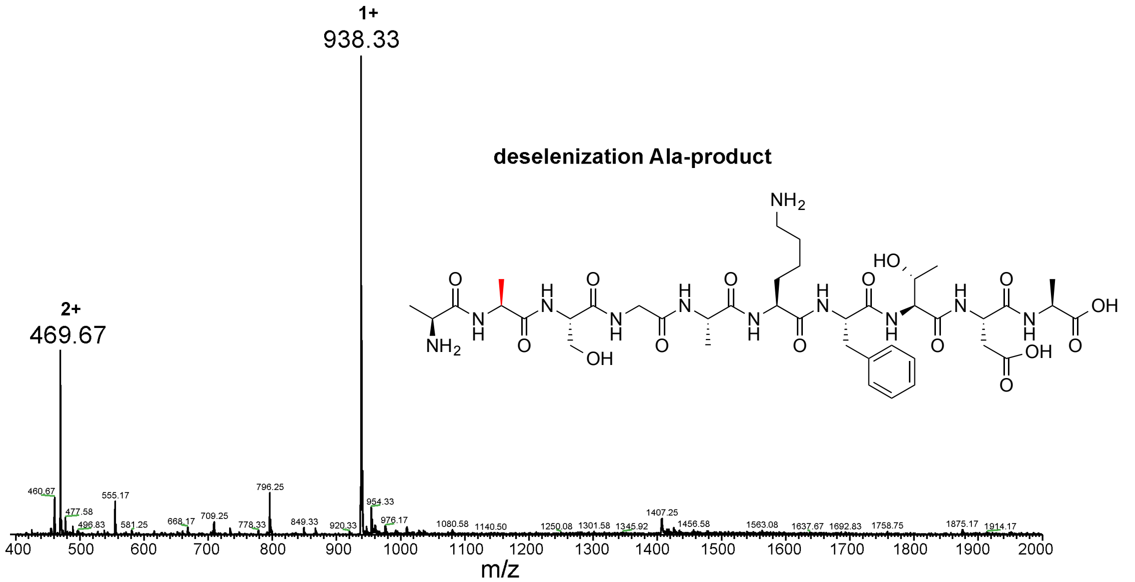

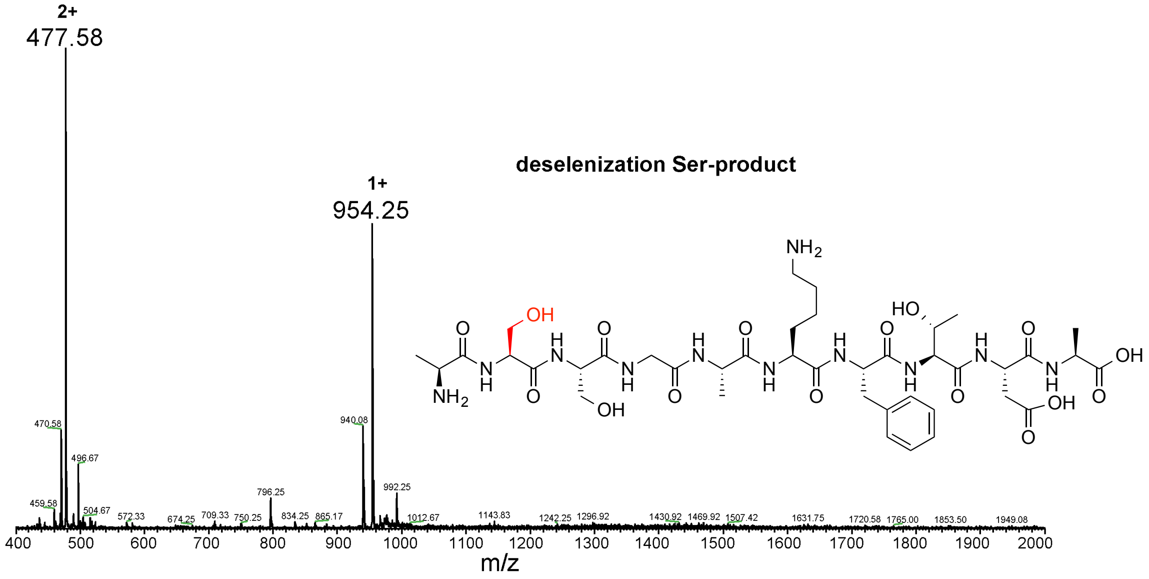

Deselenization Ala-product (A**A**SGAKFTDA): Mass calc. 937.45, found 937.34

Deselenization Ser-product (A**S**SGAKFTDA) formed under aerobic conditions: Mass calc. 953.45, found: 953.21

Peptide 2 deselenization products

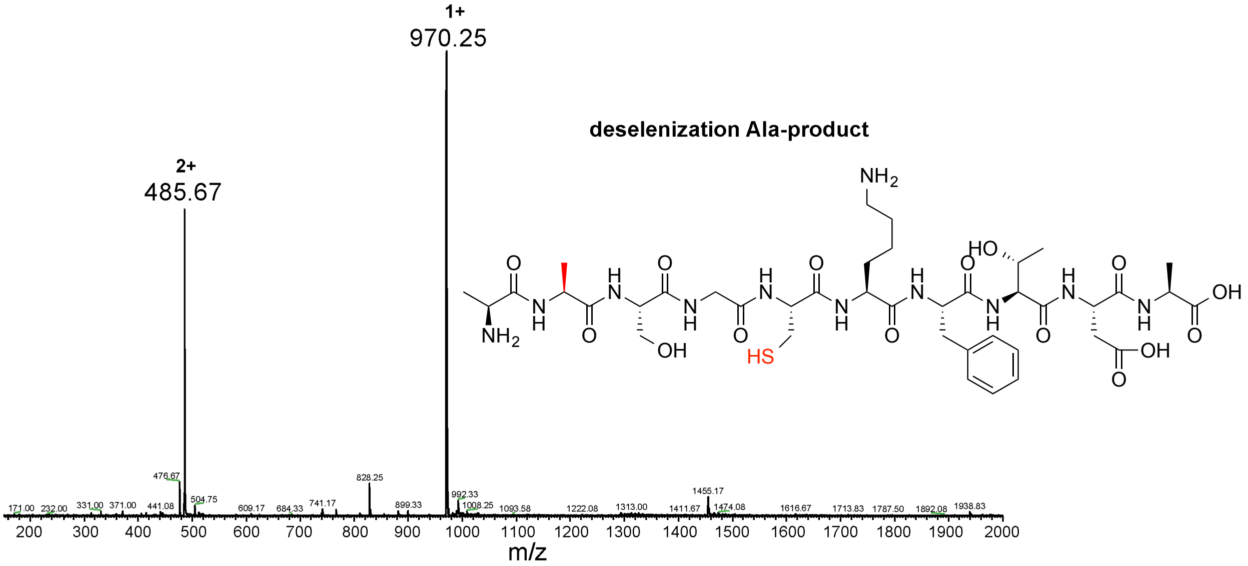

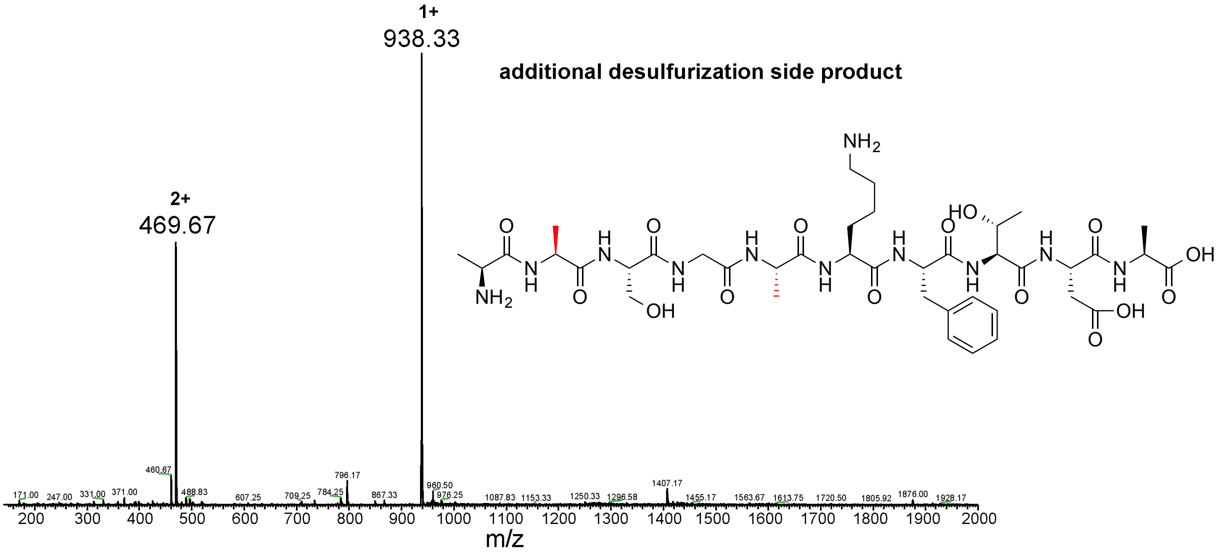

Deselenization Ala-product (A**A**SG**C**KFTDA): Mass calc. 969.42, found 969.30

Additional desulfurization side-product (A**A**SG**A**KFTDA): Mass calc. 937.45, found 937.34

Peptide 3 deselenization product

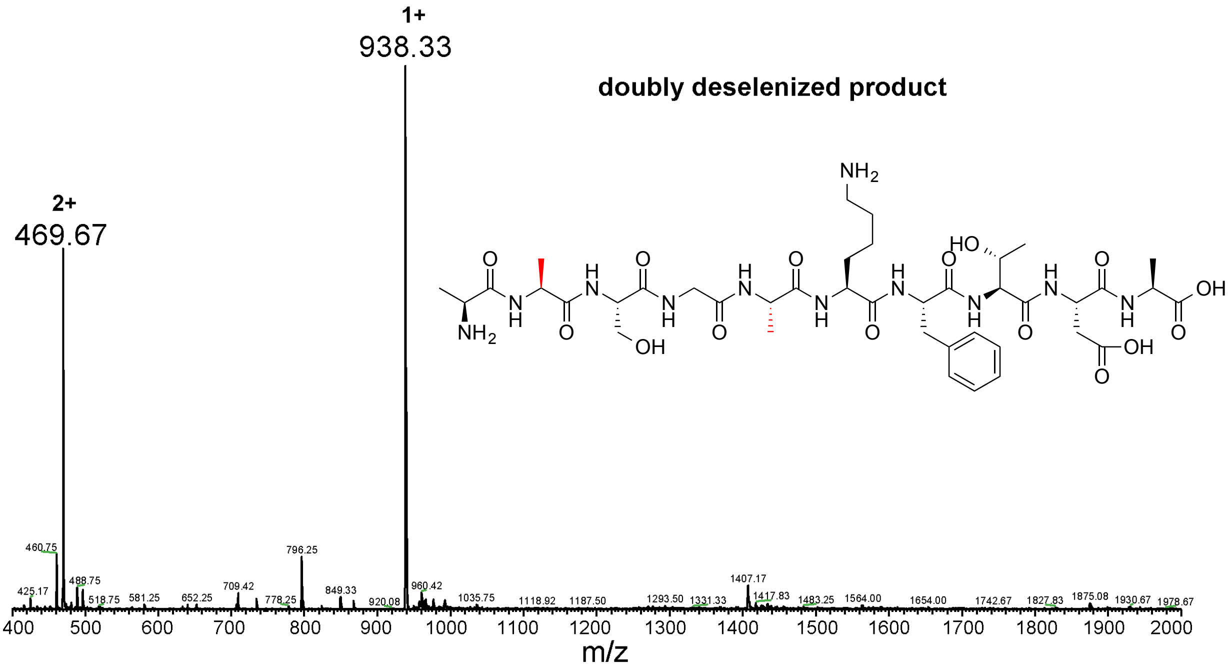

Doubly deselenization product **(**A**A**SG**A**KFTDA): Mass calc. 937.45, found 937.34

SI Figures

Figure S1: ^{1}H-NMR of Selenocystine

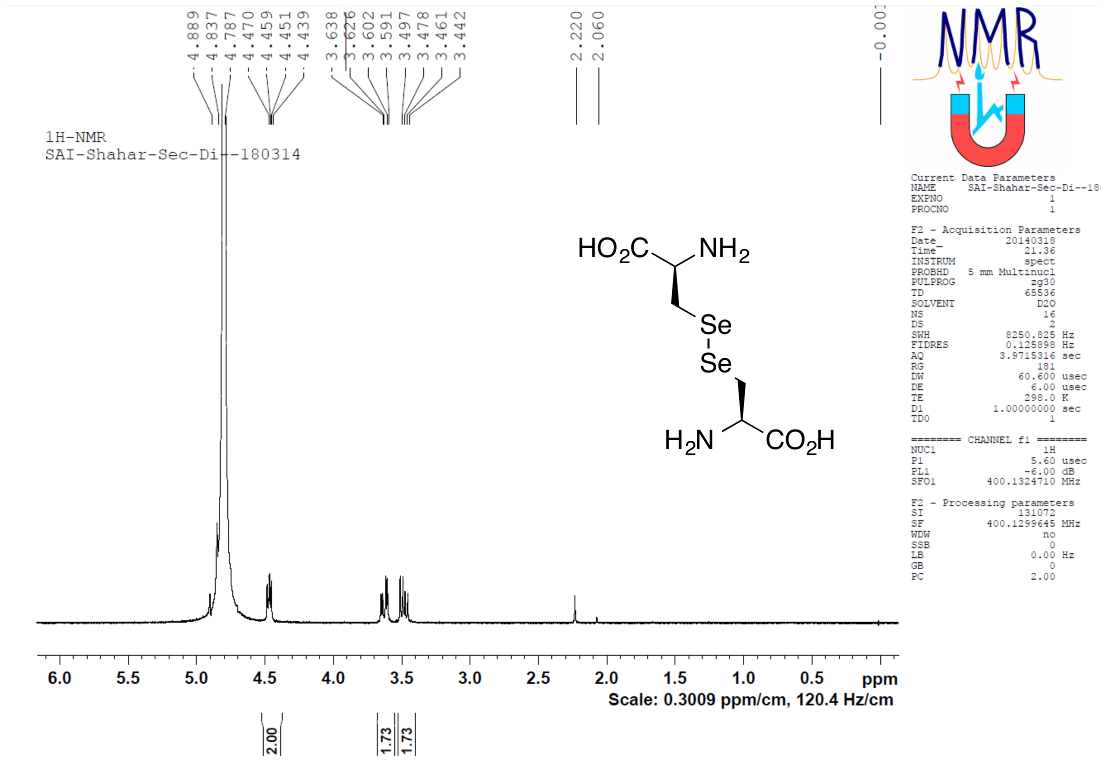

Figure S2: ^{1}H-NMR of Selenocystine + TCEP after 5 min

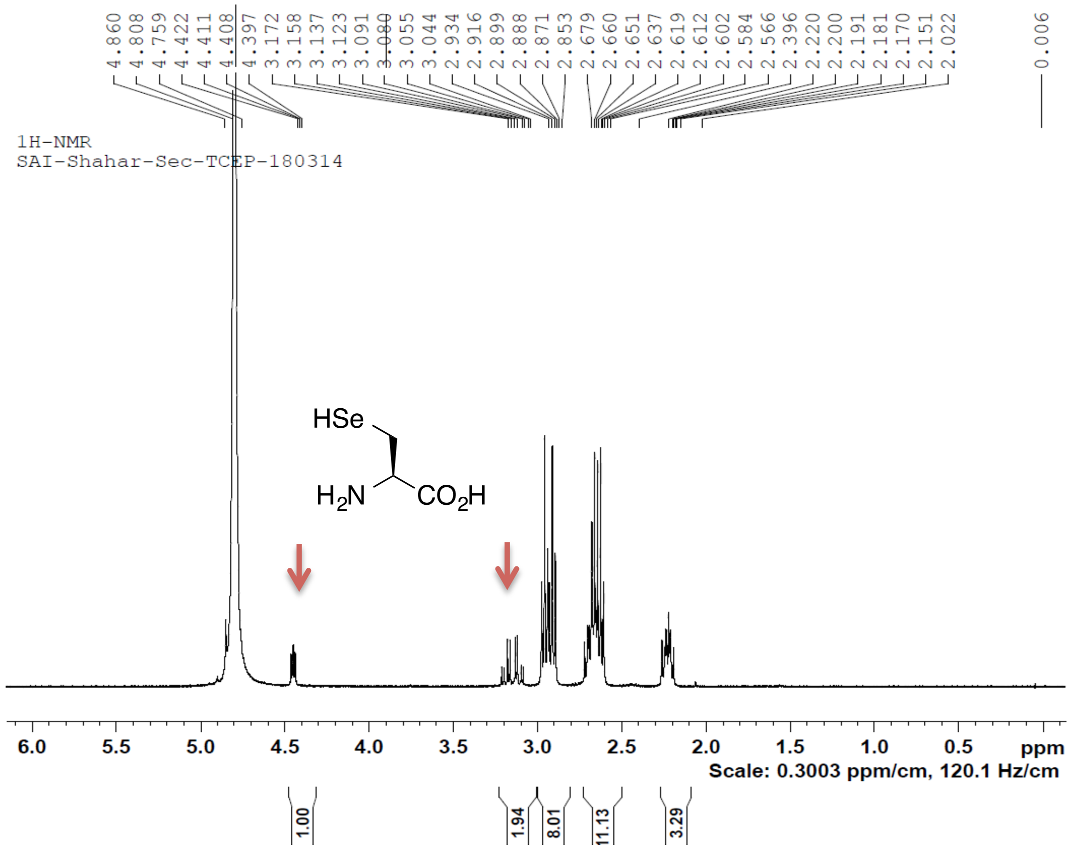

Figure S3: ^{1}H-NMR of Selenocystine + TCEP for 30 min

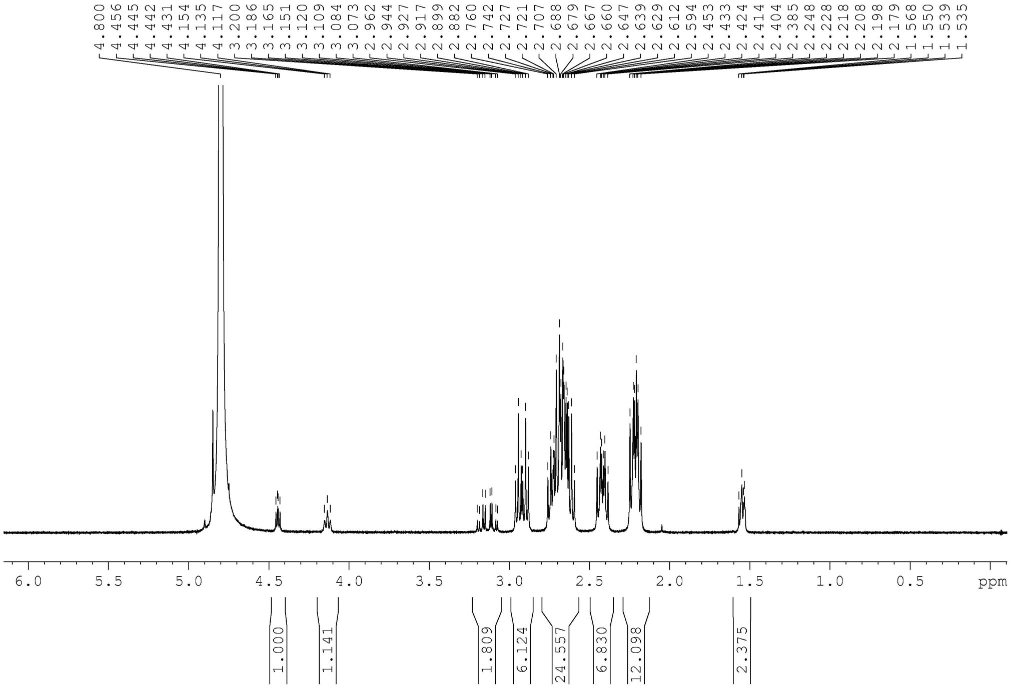

Figure S4: ^{1}H-NMR of Selenocystine + TCEP for 24 h

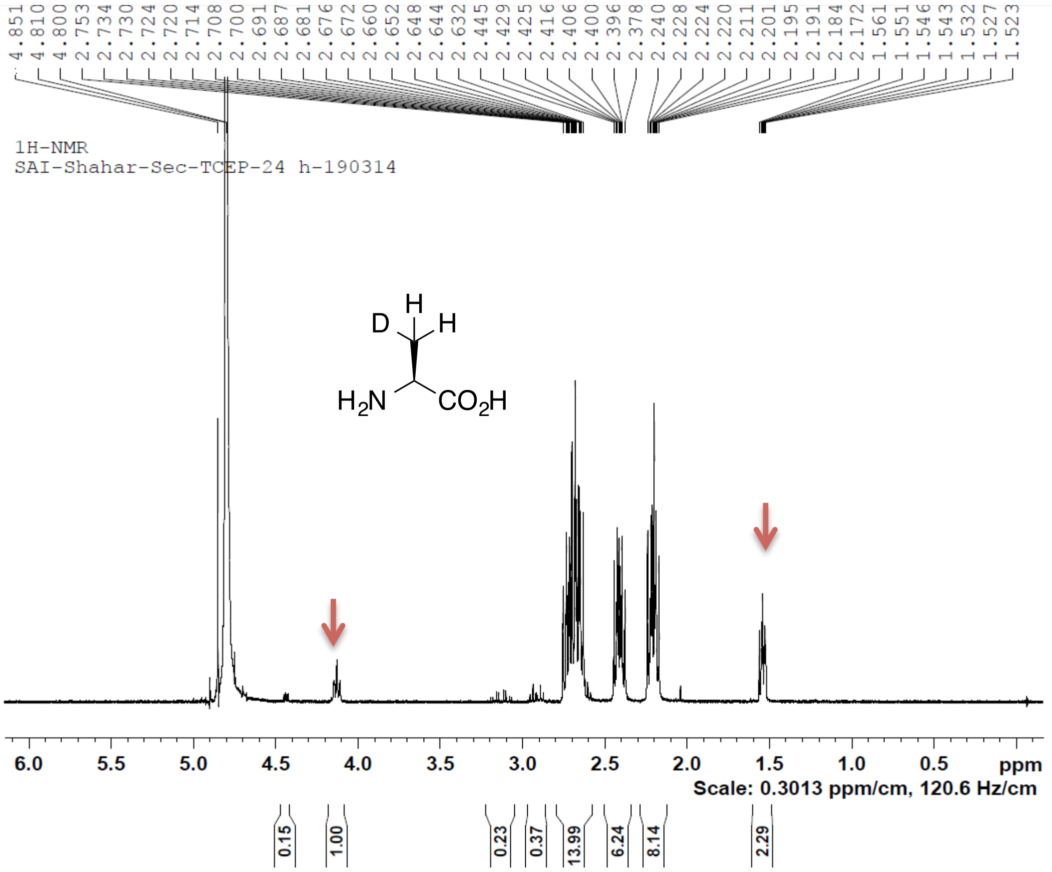

Figure S5: ^{1}H-NMR of TCEP

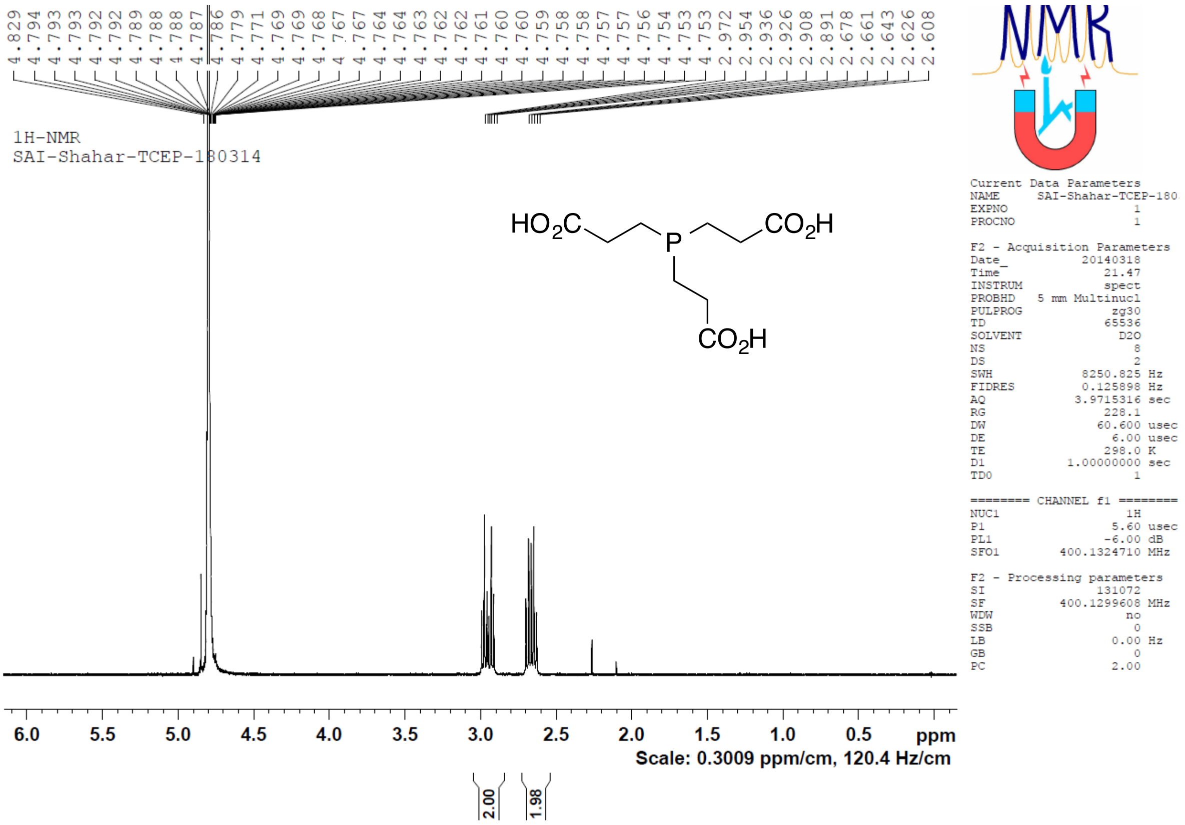

Figure S6: TCEP in solution ^{1}H-NMR – phosphorus coupled ^{1}H-NMR – phosphorus decoupled

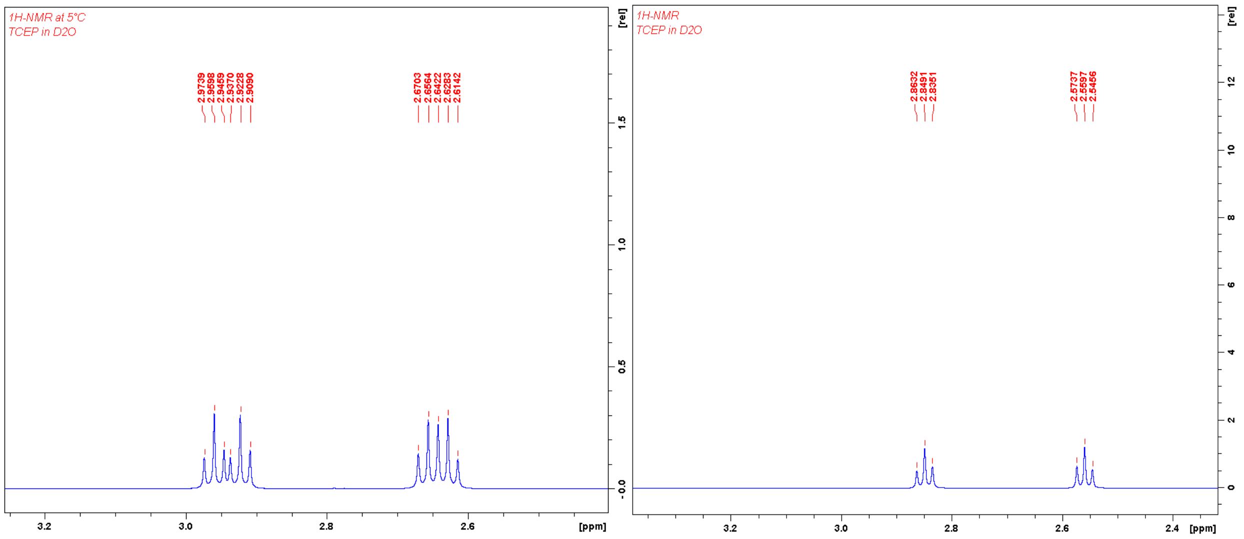

Figure S7: ^{1}H-NMR of synthetic TCEP=Se.

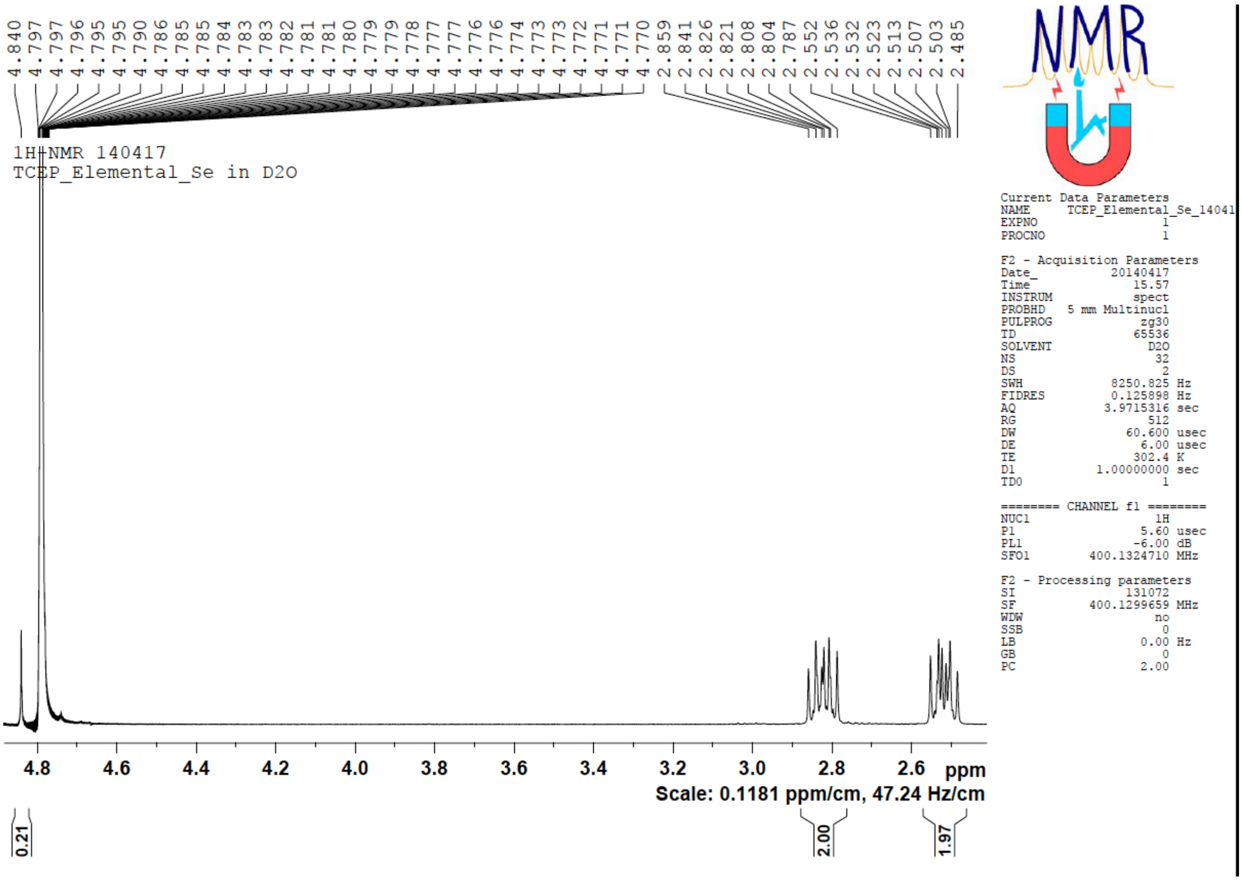

Figure S8: The NMR tube of deselenization reaction after two weeks

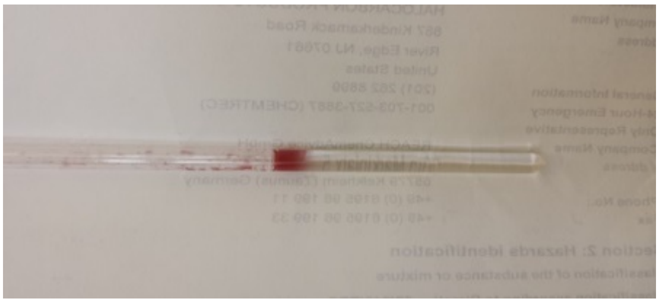

Figure S9: ^{31}P-NMR of TCEP.

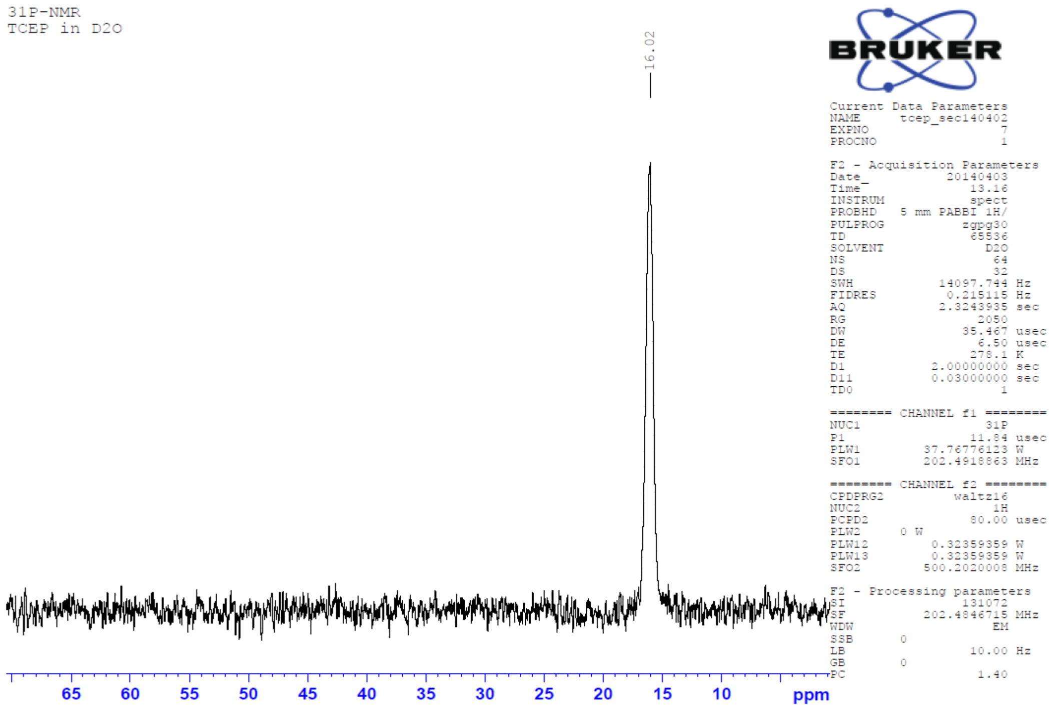

Figure S10: ^{31}P-NMR of synthetic TCEP=Se. The satellite peaks correspond to $^{1}J\,^{77}Se-^{31}P$ coupling. The ^{31}P-NMR result confirms the presence of only one phosphorus compound – TCEP=Se.

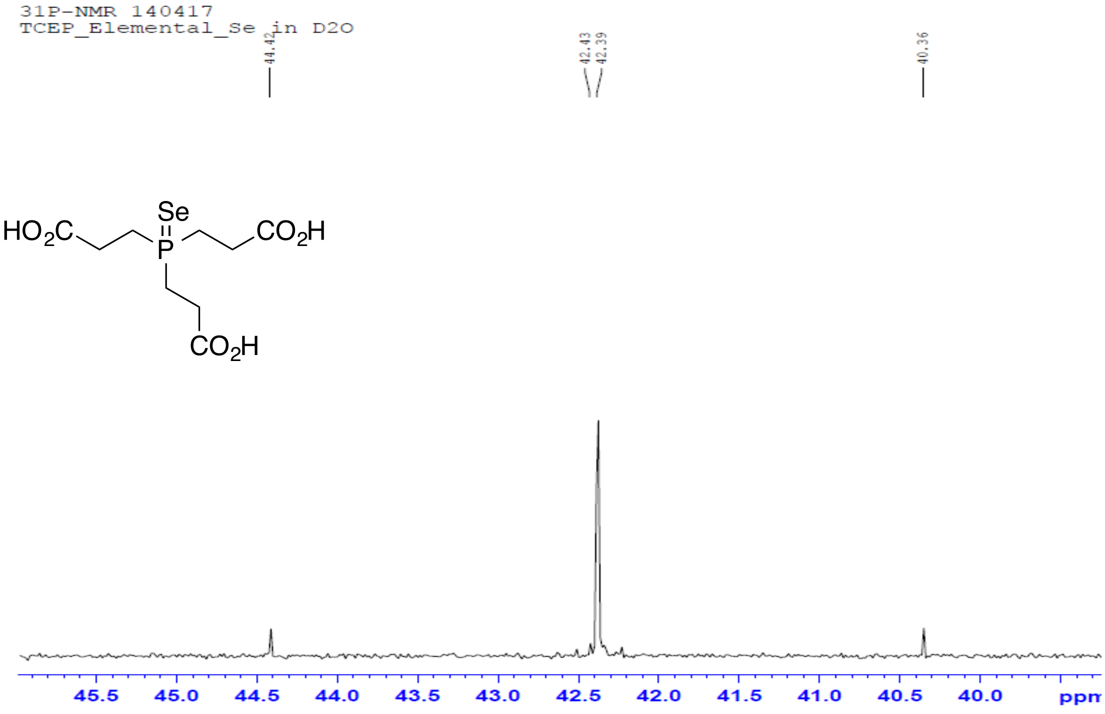

Figure S11: ^{31}P-NMR of Selenocystine + TCEP for 5 min; TCEP at 15.7 ppm, TCEP=O at 56.8 ppm)

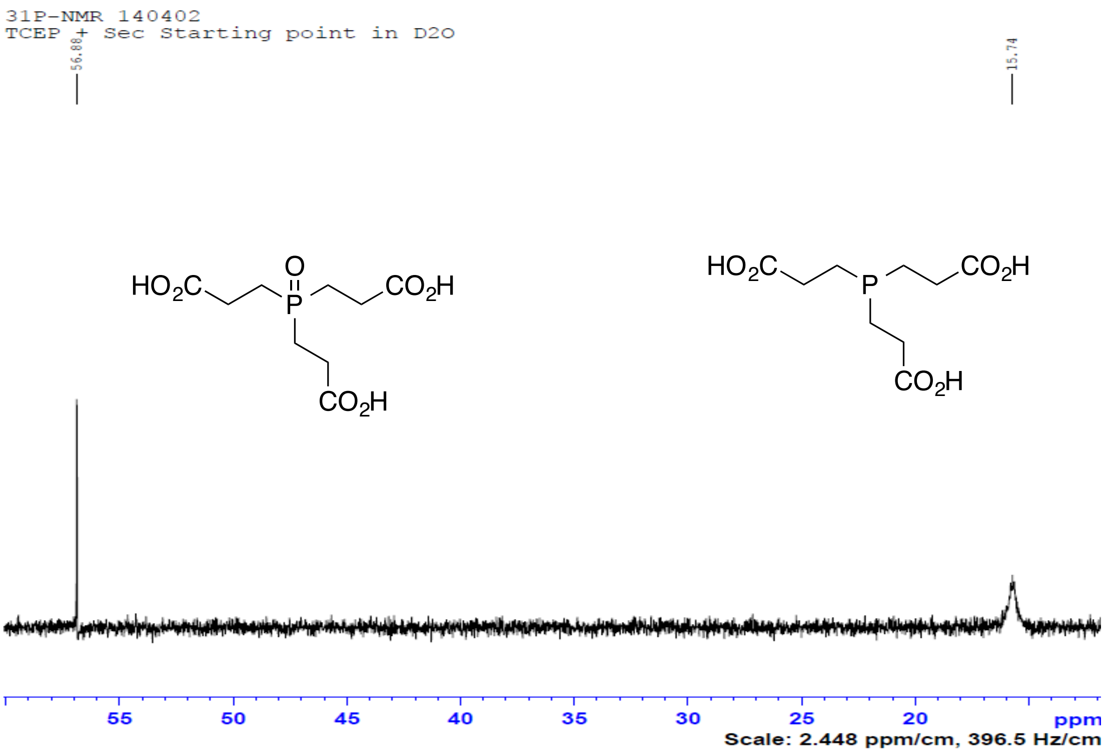

Figure S12: ^{31}P-NMR of Selenocystine + TCEP for 24 h. TCEP at 15.7 ppm, TCEP=O at 56.8 ppm; TCEP=Se at 42.3 ppm confirmed with the synthetic TCEP=Se.

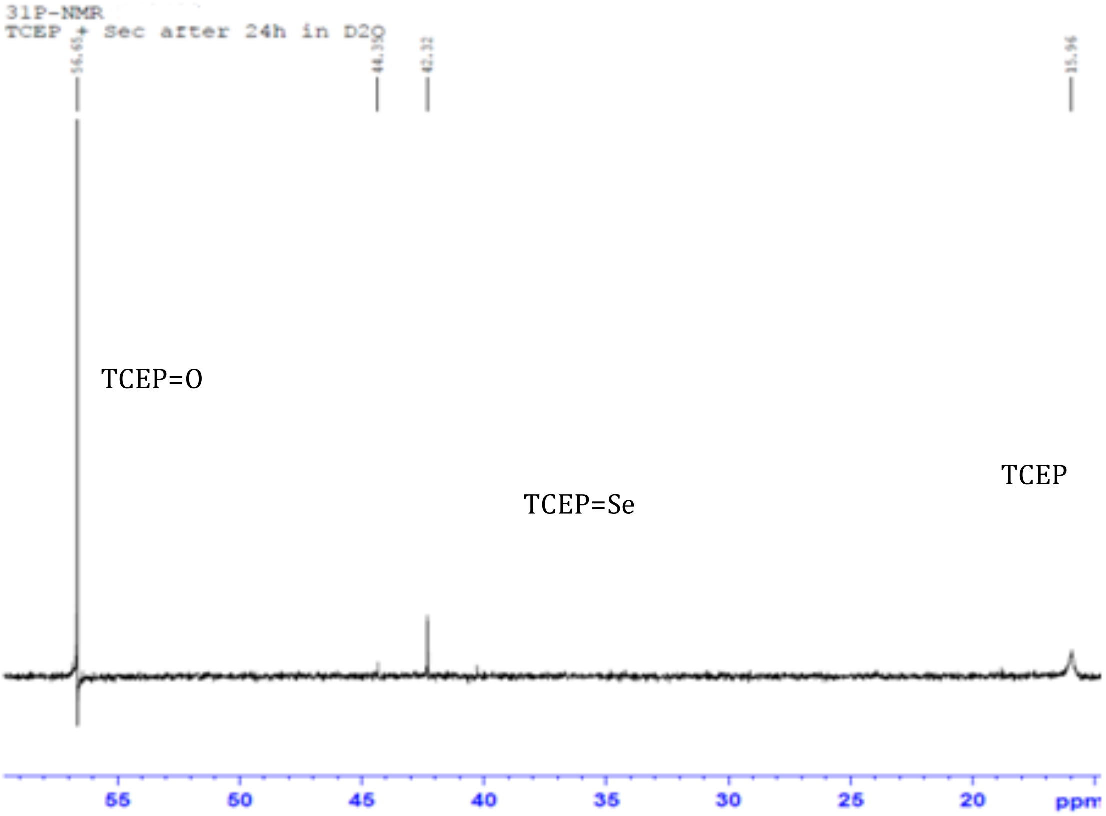

Figure S13: 2 mM peptide **1**, 200 equiv. TCEP, 100 mM PB, pH 5, aerobic or anaerobic, ambient light, 23°C after only 1 min.

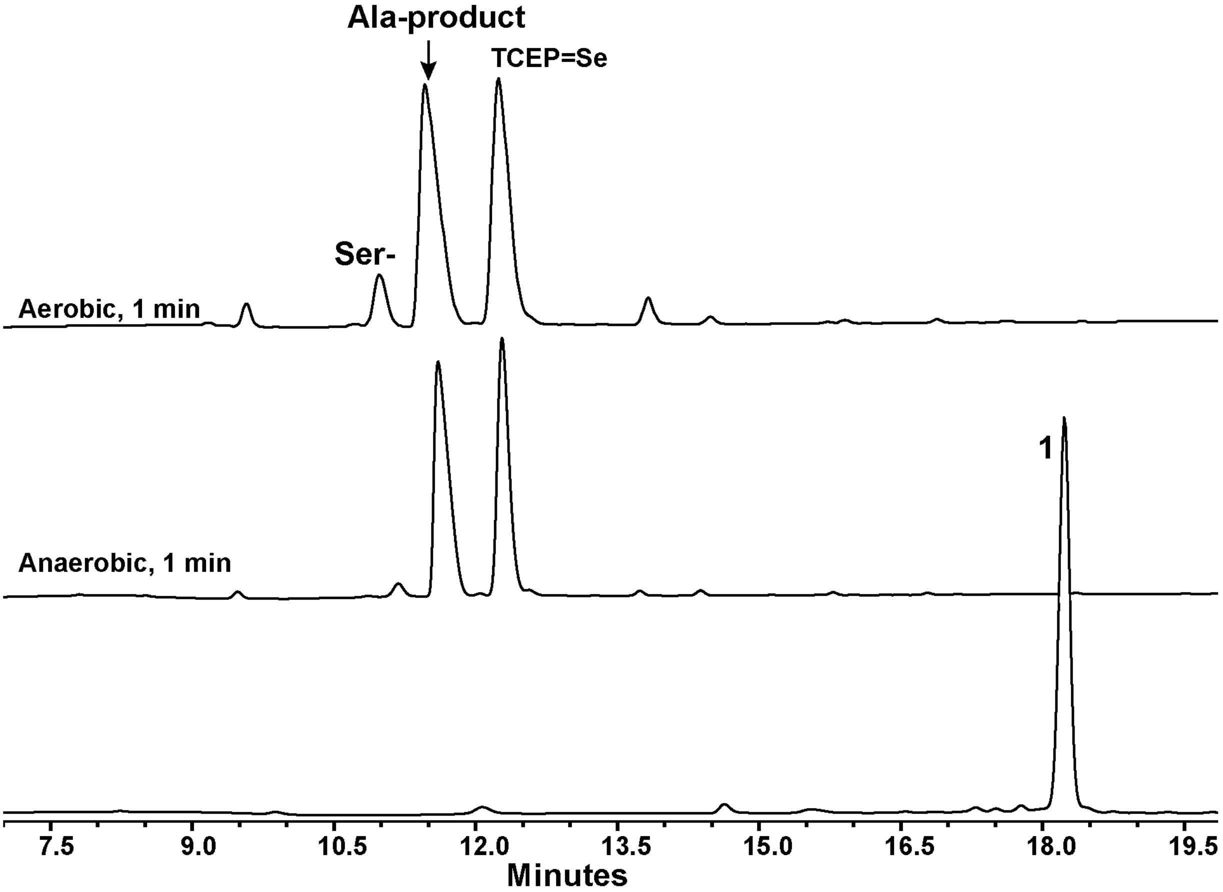

Figure S14: 2 mM peptide **5**, 200 equiv. TCEP, 100 mM PB, pH 5, aerobic, ambient light, 23°C

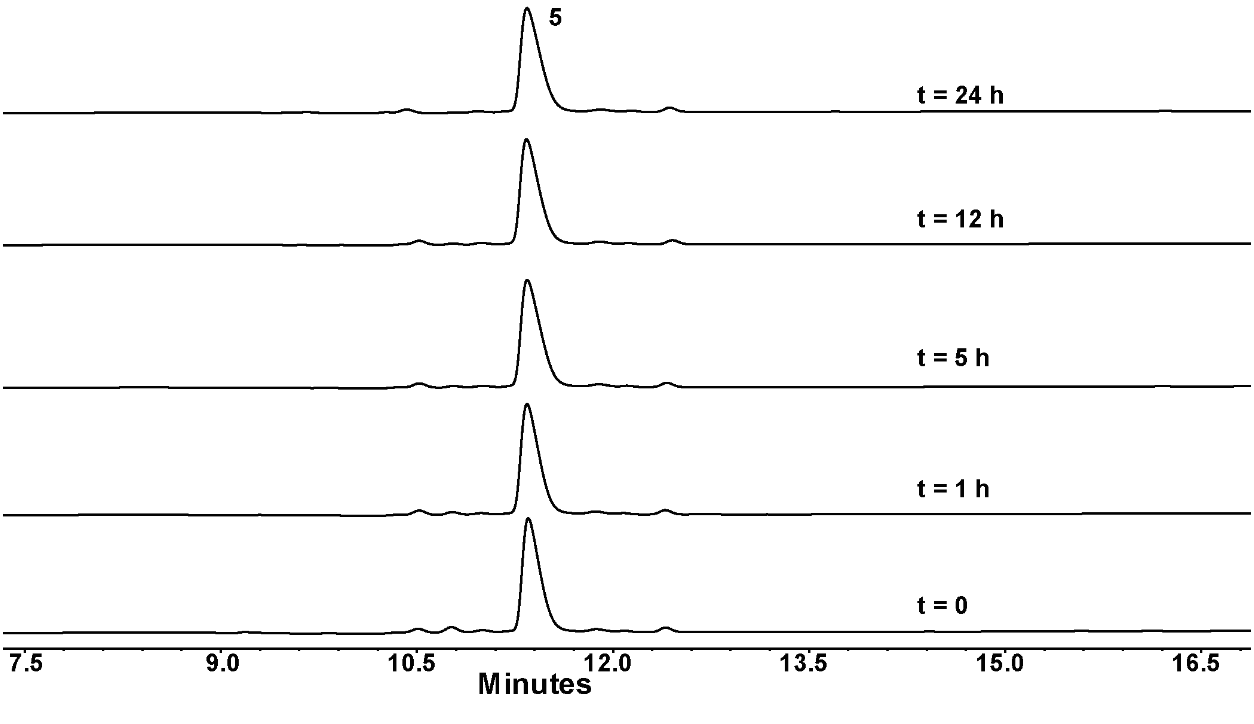

Figure S15: 2 mM peptide **1**, 10 equiv. TCEP with or without 10 equiv. VA-044, anaerobic, ambient light at 23°C.

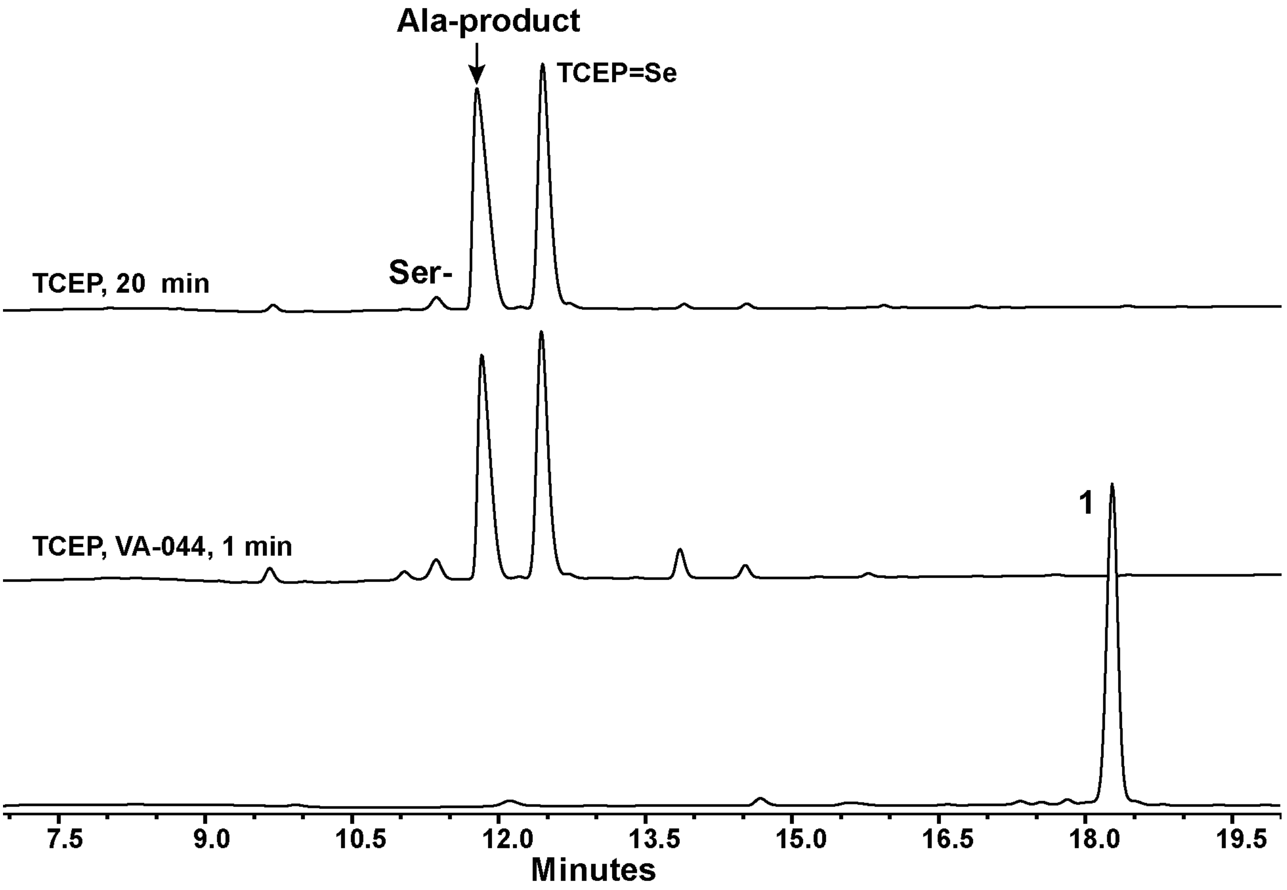

Figure S16: 2 mM peptide **5**, 200 equiv. TCEP, 10 equiv. VA-044, 100 mM PB, pH 5, anaerobic, ambient light, 37°C

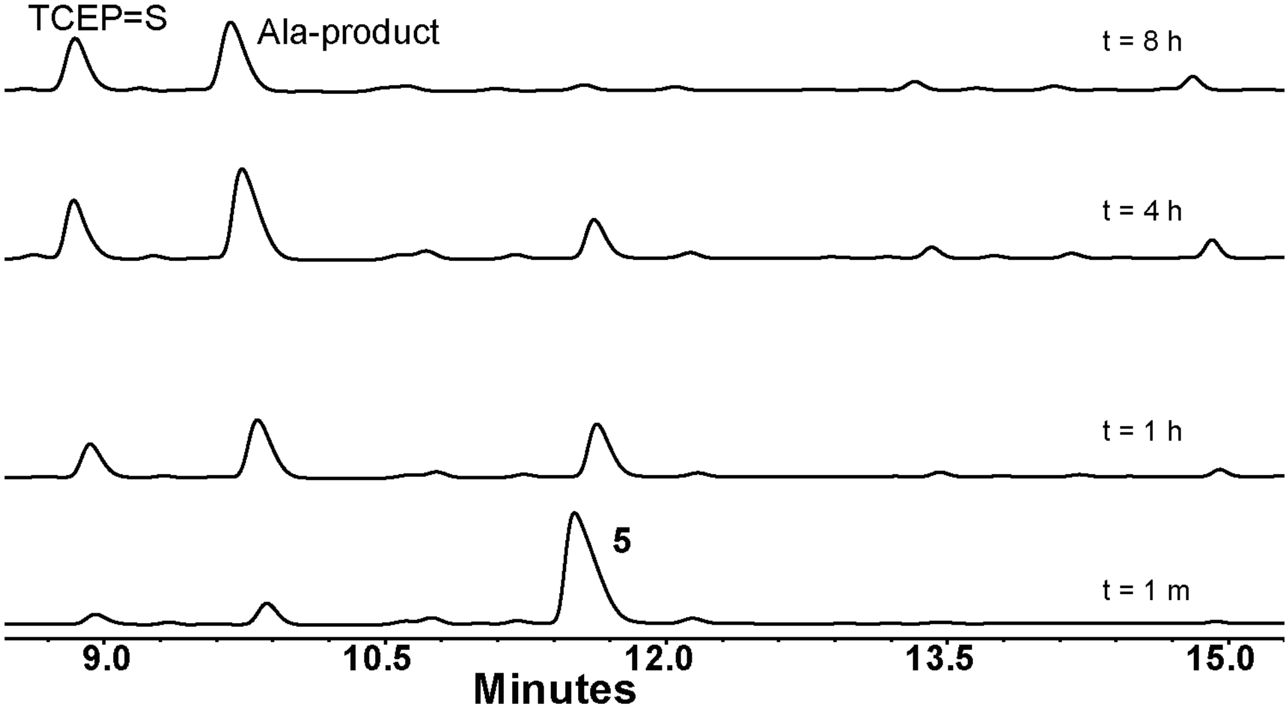

Figure S17: 2 mM peptide **1**, 10 equiv. TCEP, 50 equiv. sodium ascorbate, 100 mM PB, pH 5, aerobic, ambient light, 23°C, minor products were observed after 12 h.

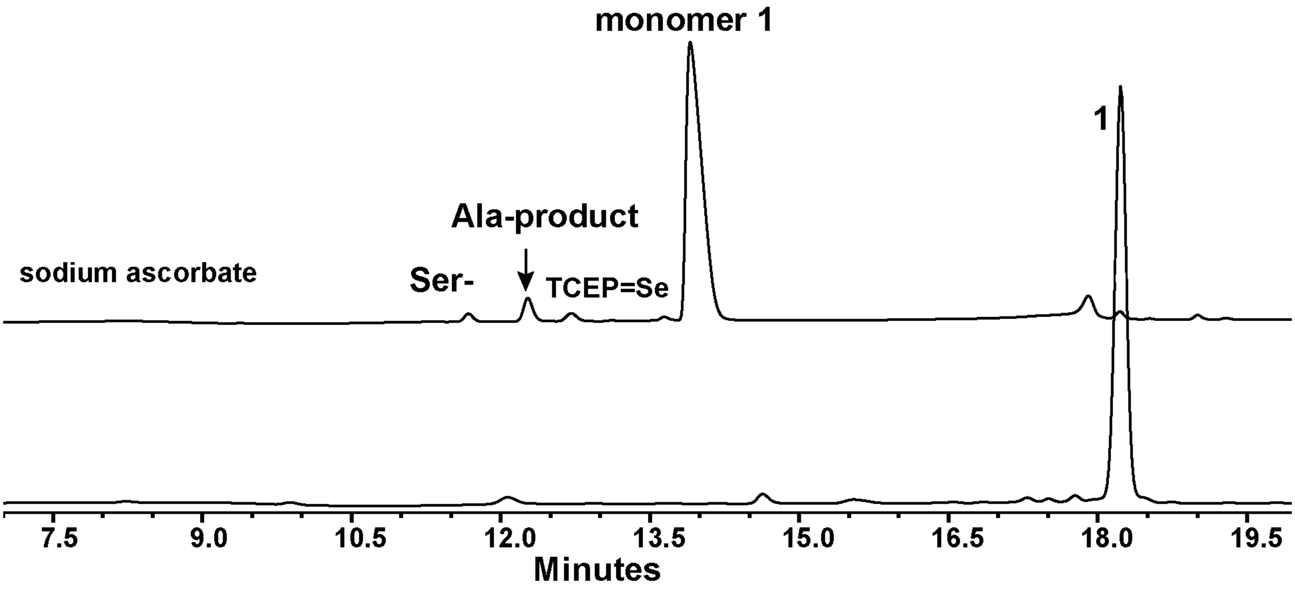

Figure S18: 2 mM peptide **1**, 50 equiv. TCEP, 200 equiv. TCEP=Se, 100 mM PB, pH 5, aerobic, ambient light, 23°C. TCEP=Se does not inhibit the deselenization reaction (1, 10, 20, 30 min).

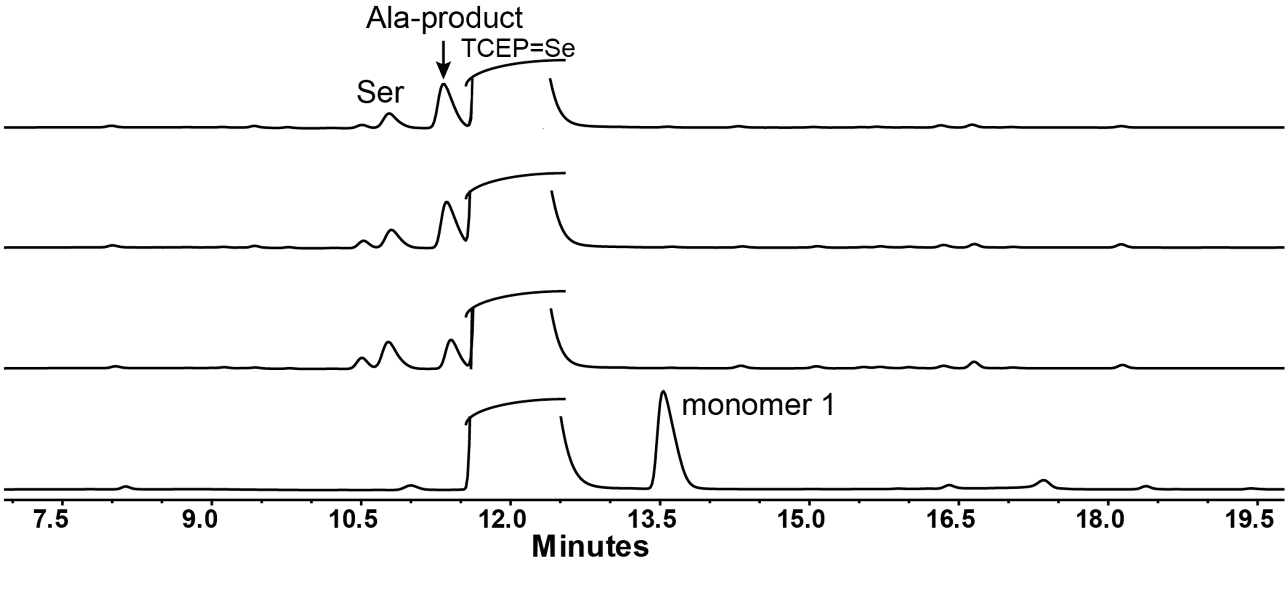

Figure S19: 2 mM peptide **2**, 2 equiv. TCEP, 100 mM PB, pH 5, anaerobic, ambient light, 23°C. At time zero we observe dimer isomers of peptide **2** (small peaks). The reduced form of **2** sticks to the column (R_t =14.5 min). Peak § is the additional desulfurization side-product.

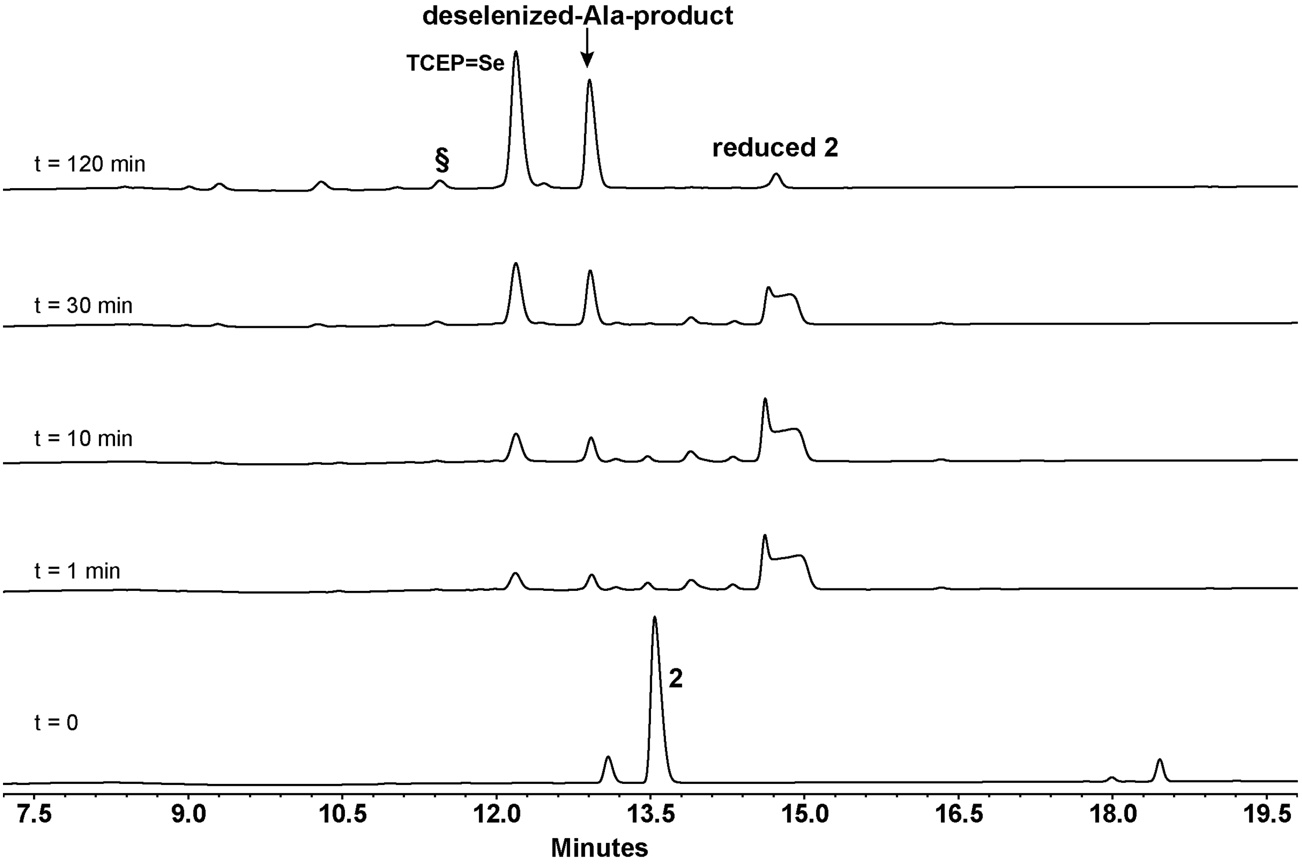

Figure S20: 2 mM peptide **3**, 200 equiv. TCEP, 2 equiv. VA-044, pH 5, anaerobic chamber, ambient light, 23°C. The reaction proceeded smoothly to completion in 30 min. Peaks # and * are the two dimer isomers of **3**.

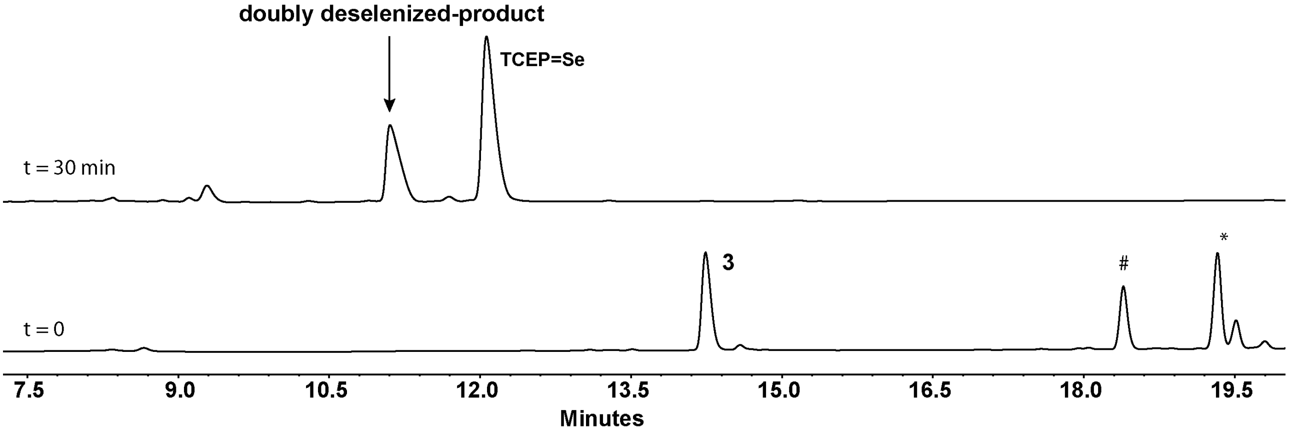

Figure S21: 2 mM peptide **7**, required 200 equiv. TCEP, with or without 10 equiv. VA-044, 100 mM PB, pH 5, aerobic at ambient light and 37°C, to give the doubly desulfurized product after 8 h.

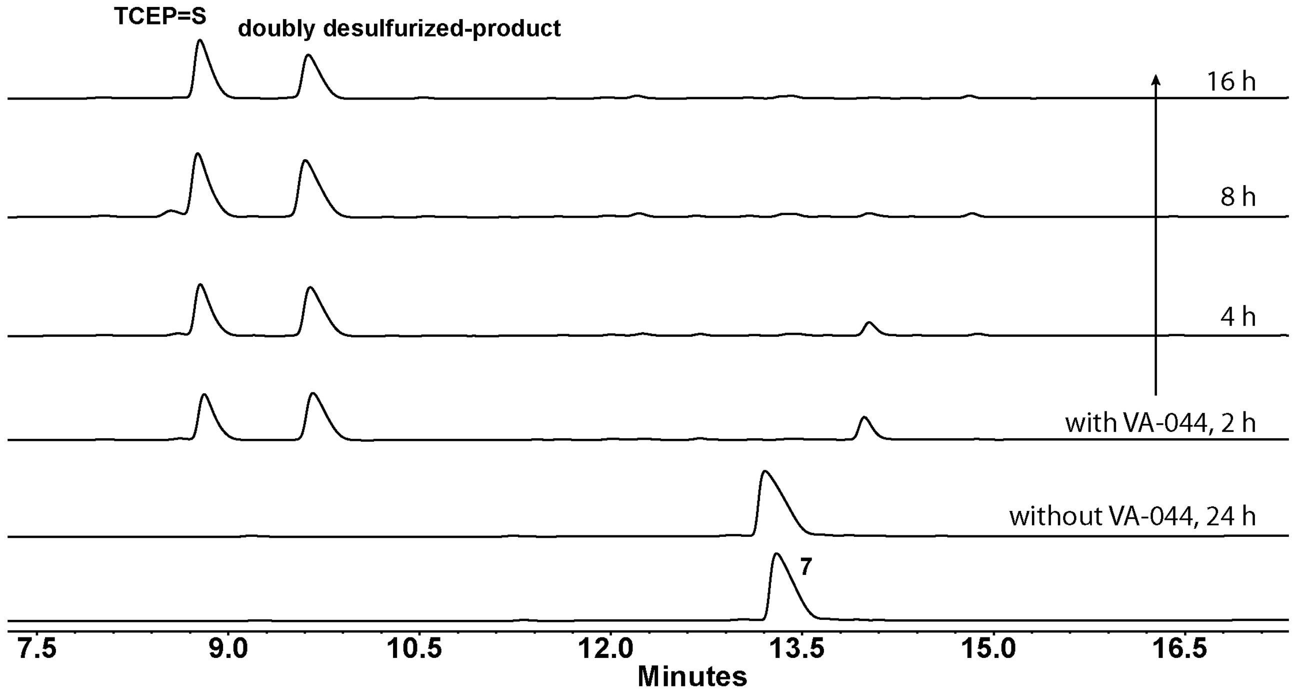

Figure S22: BPTI C5U (peptide **4**) synthesis has been published recently.[6] The selective deselenization of BPTI C5U (58 amino acids long containing 5 Cys and 1 Sec) was completed in 4 h giving the desired Ala-product BPTI C5A. ESI-MS for the BPTI C5U (calc. 6546.4 Da, found 6546.1 Da) and the deselenization product BPTI C5A (calc. 6467.5 Da, found 6467.4 Da) are shown. Difference of 79 Da indicates the Sec conversion into Ala.

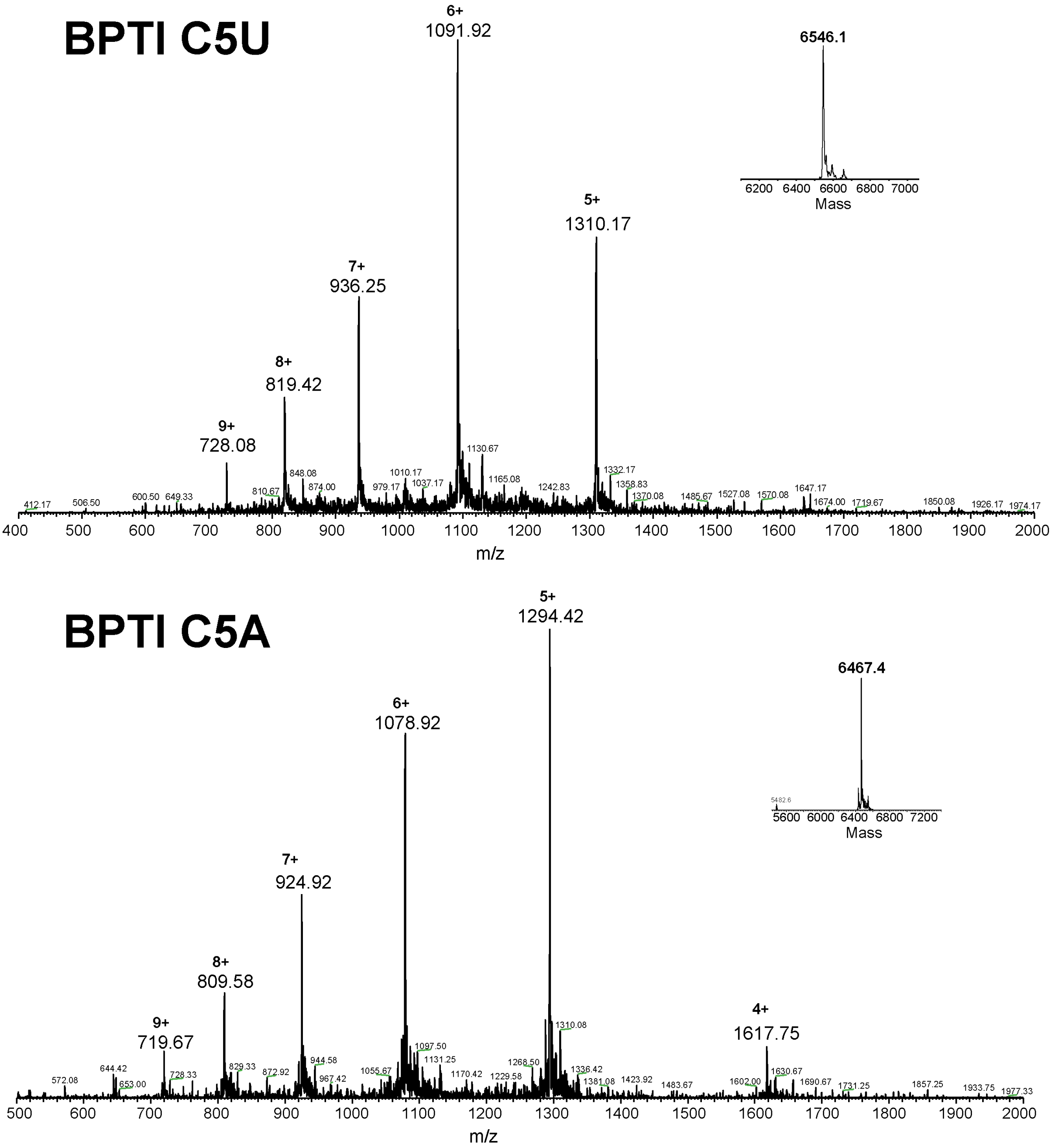

Figure S23: 2 mM peptide **1**, 50 equiv. TCEP, 100 mM PB, pH 5, aerobic + air flow 10 min, ambient light, 23°C.

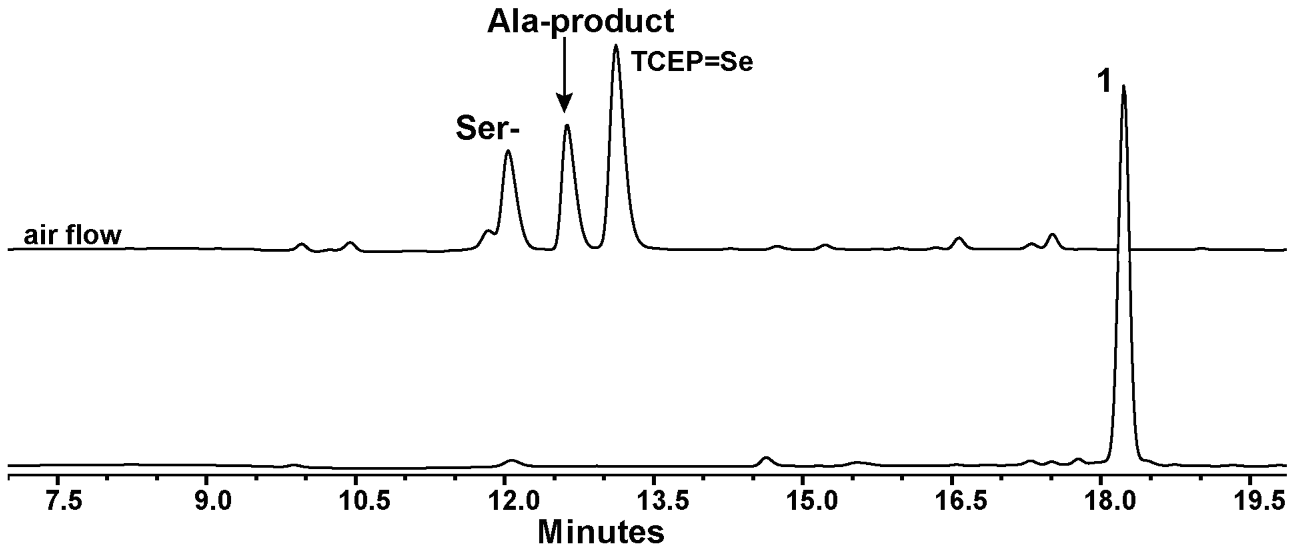

References

1. N. Metanis, E. Keinan and P. E. Dawson, *J. Am. Chem. Soc.*, 2006, **128**, 16684-16691.
2. M. D. Gieselman, L. L. Xie and W. A. van der Donk, *Org. Lett.*, 2001, **3**, 1331-1334.
3. M. Schnolzer, P. Alewood, A. Jones, D. Alewood and S. B. H. Kent, *International Journal of Peptide Research and Therapeutics*, 2007, **13**, 31-44.
4. Y. Kajihara, Y. Kanemitsu, M. Nishihara, R. Okamoto and M. Izumi, *J. Pept. Sci.*, 2014, **20**, 958-963.
5. S. Flemer and R. J. Hondal, *Biopolymers*, 2011, **96**, 439-439.
6. N. Metanis and D. Hilvert, *Chem. Sci.*, 2015, **6**, 322-325.
